# Reaction of pyranose dehydrogenase from *Agaricus meleagris* with its carbohydrate substrates

**DOI:** 10.1111/febs.13417

**Published:** 2015-09-11

**Authors:** Michael M.H. Graf, Jeerus Sucharitakul, Urban Bren, Dinh Binh Chu, Gunda Koellensperger, Stephan Hann, Paul G. Furtmüller, Christian Obinger, Clemens K. Peterbauer, Chris Oostenbrink, Pimchai Chaiyen, Dietmar Haltrich

**Affiliations:** ^1^ Food Biotechnology LaboratoryDepartment of Food Science and TechnologyUniversity of Natural Resources and Life Sciences Vienna (BOKU)Austria; ^2^ Department of BiochemistryFaculty of DentistryChulalongkorn UniversityBangkokThailand; ^3^ Institute of Molecular Modeling and SimulationUniversity of Natural Resources and Life Sciences Vienna (BOKU)Austria; ^4^ Laboratory for Physical Chemistry and Chemical ThermodynamicsFaculty of Chemistry and Chemical TechnologyUniversity of MariborSlovenia; ^5^ Division of Analytical ChemistryDepartment of ChemistryUniversity of Natural Resources and Life Sciences Vienna (BOKU)Austria; ^6^ School of Chemical EngineeringDepartment of Analytical ChemistryHanoi University of Science and TechnologyHanoiVietnam; ^7^ Institute of Analytical ChemistryFaculty of ChemistryUniversity of ViennaAustria; ^8^ Division of BiochemistryDepartment of ChemistryUniversity of Natural Resources and Life Sciences Vienna (BOKU)Austria; ^9^ Department of Biochemistry and Center of Excellence in Protein Structure and FunctionFaculty of ScienceMahidol UniversityBangkokThailand

**Keywords:** *Agaricus meleagris* pyranose dehydrogenase, enzyme kinetics, flavin‐dependent oxidoreductase, glucose–methanol–choline family of oxidoreductases, molecular dynamics simulations

## Abstract

Monomeric *Agaricus meleagris* pyranose dehydrogenase (*Am* PDH) belongs to the glucose–methanol–choline family of oxidoreductases. An FAD cofactor is covalently tethered to His103 of the enzyme. *Am* PDH can double oxidize various mono‐ and oligosaccharides at different positions (C1 to C4). To study the structure/function relationship of selected active‐site residues of *Am* PDH pertaining to substrate (carbohydrate) turnover in more detail, several active‐site variants were generated, heterologously expressed in *Pichia pastoris*, and characterized by biochemical, biophysical and computational means. The crystal structure of *Am* PDH shows two active‐site histidines, both of which could take on the role as the catalytic base in the reductive half‐reaction. Steady‐state kinetics revealed that His512 is the only catalytic base because H512A showed a reduction in (*k*_cat_/*K*_M_)_glucose_ by a factor of 10^5^, whereas this catalytic efficiency was reduced by two or three orders of magnitude for His556 variants (H556A, H556N). This was further corroborated by transient‐state kinetics, where a comparable decrease in the reductive rate constant was observed for H556A, whereas the rate constant for the oxidative half‐reaction (using benzoquinone as substrate) was increased for H556A compared to recombinant wild‐type *Am* PDH. Steady‐state kinetics furthermore indicated that Gln392, Tyr510, Val511 and His556 are important for the catalytic efficiency of PDH. Molecular dynamics (MD) simulations and free energy calculations were used to predict d‐glucose oxidation sites, which were validated by GC‐MS measurements. These simulations also suggest that van der Waals interactions are the main driving force for substrate recognition and binding.

**Abbreviations**
*Ab*PDH: pyranose dehydrogenase from *Agaricus bisporus*
*Am*PDH: pyranose dehydrogenase from *Agaricus meleagris*
ARA: l‐arabinose
BQ: 1,4‐benzoquinone
CV: column volume
DCIP: 2,6‐dichlorophenol‐indophenol
ECD: electronic circular dichroism
Fc^+^: ferrocenium ion
GAL: d‐galactose
GC‐CI‐QToFMS: gas chromatography combined with chemical ionization time of flight mass spectrometry
GC‐EI‐MS: gas chromatography and electron ionization mass spectrometry
GLC: d‐glucose
His_6_‐tag: hexahistidine‐tag
IMAC: immobilized metal affinity chromatography
MD: molecular dynamics
MGP: methyl‐α‐d‐glucopyranoside
PDB: Protein Data Bank
POx: pyranose 2‐oxidase
TCA: trichloroacetic acid

## Introduction

The oxidoreductase pyranose dehydrogenase from the litter‐decomposing fungus *Agaricus meleagris* (*Am*PDH; EC 1.1.99.29; PDB code: 4H7U) is a glycoprotein of approximately 65 kDa carrying a monocovalently linked FAD cofactor 1. The level of glycosylation depends on the source of PDH, ranging from approximately 7% (natural source *A. meleagris*) 2 to 30% (overexpressed in *Pichia pastoris*) 3. Together with aryl‐alcohol oxidase 4, choline oxidase 5, glucose 1‐oxidase 6 and pyranose 2‐oxidase (POx) 7, amongst others, *Am*PDH belongs to the structural family of glucose–methanol–choline oxidoreductases 1, 8, 9. PDH was initially identified and studied in *Agaricus bisporus* 10 and *Macrolepiota rhacodes* 11; however, PDH from *A. meleagris* 12 is the best characterized to date, with its crystal structure solved to a resolution of 1.6 Å 13. In accordance with other flavin‐dependent oxidoreductases, PDH shows a reaction mechanism consisting of two half‐reactions. In the reductive half‐reaction, an electron donor substrate (a sugar substrate) is oxidized, whereas the flavin is reduced. In the ensuing oxidative half‐reaction, the reduced flavin is re‐oxidized by a second electron‐acceptor substrate. *Am*PDH can oxidize a wide range of different carbohydrates, and d‐glucose (GLC) is one of its favoured substrates as judged by the catalytic efficiency 2. Studies on the oxidized sugar products revealed that PDH is able to double oxidize mono‐ and oligosaccharides at C1–C4 12, 14, 15, 16. Molecular dynamics (MD) simulations suggested preferred modes of productive binding for different monosaccharide substrates, as well as an oxidation mechanism for GLC 17, 18. PDH shows very poor activity with oxygen in its oxidative half‐reaction but readily reduces electron acceptors such as various substituted quinones, redox dyes or chelated metal ions. Previous characterizations of *Am*PDH have either focused on wild‐type *Am*PDH 2, 13 or on identifying variants with increased oxygen reactivity employing a semi‐rational approach coupled with high‐throughput screening 19, 20.

**Figure 1:**
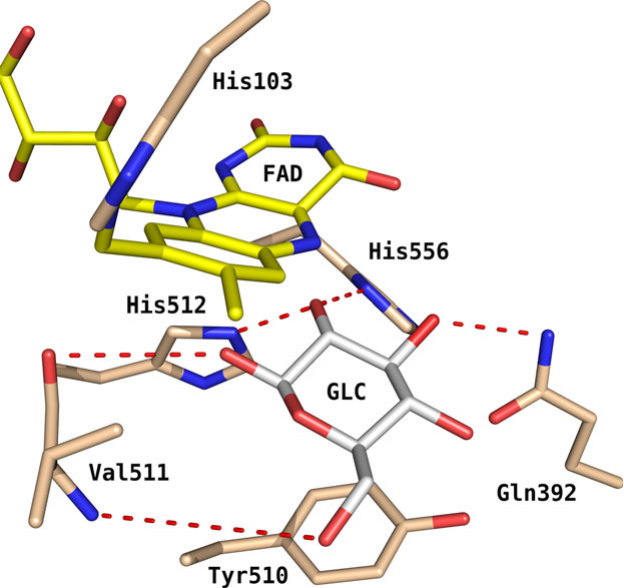
Active site of *Am* PDH (PDB code: 4H7U) with GLC bound according to pose A (i.e. oriented for oxidation at C2). GLC coordinates from the closely related *Tm* POx (PDB code: 3PL8) were grafted into *Am* PDH after superimposing the X‐ray structures of both enzymes. Atom‐colouring scheme: carbon (beige, protein; yellow, FAD; white, ligand), nitrogen (blue) and oxygen (red). Red dashes represent important hydrogen bonds between GLC and *Am* PDH found during molecular dynamics simulations 17 and docking 13 in previous studies. Image generated using pymol (http://www.pymol.org/).

The present study represents the first comprehensive rational investigation of several active‐site residues in *AmPDH* (Fig. 1) 13, 17, 19. Previous computational studies on *Am*PDH suggested that both active‐site histidines (His512 and His556) potentially act as catalytic base, and thus are responsible for C2 and C3 oxidation 17, 19, respectively. These studies also showed that residues Gln392, Tyr510 and Val511 might play a role in GLC binding and affect the site of GLC oxidation 17. To test these hypotheses, active‐site variants Q392A, Y510A, V511F, V511W, H512A, H556A and H556N were generated by site‐directed mutagenesis. Additionally, the covalent linkage between His103 and FAD was disrupted in variant H103A to probe the influence of the covalent bond on the enzyme's properties. These variants were characterized both biochemically and biophysically by application of a broad set of methods.

## Results and Discussion

### Cloning, expression and purification

We chose six active‐site residues as targets for mutagenesis (Table S1) and structure/function studies based on the crystal structure of *A. meleagris* PDH 13 (*Am*PDH; PDB code: 4H7U) and on recent findings employing MD simulations 17, namely His103, Gln392, Tyr510, Val511, His512 and His556 (Fig. 1). The computational studies indicated that these residues might be involved in substrate binding (Gln392, Tyr510, Val511, His556) or catalysis (His512, His556). We generated the variants V511F and V511W (to probe whether the backbone oxygen atom of Val511 supports substrate binding via hydrogen‐bond formation) 17, as well as H512A and H556A (for testing the function of these histidines as catalytic bases). Additionally, the variant H556N was produced because the catalytically related enzyme pyranose 2‐oxidase (which uses O_2_ as electron acceptor) from *Trametes multicolor* (*Tm*POx) carries an asparagine at position 593 7, 21 that corresponds to His556 in *Am*PDH. Other variants that were studied in an alanine‐scanning approach included H103A (removal of the covalent attachment of FAD), Q392A (removal of a hydrogen bond with bound sugar substrate) and Y510A (removal of stacking interaction with bound sugar substrate). All proteins were heterologously expressed using *P. pastoris*, which proved to be superior for the production of recombinant *Am*PDH to other microbial expression systems 3. A purification scheme based on cross‐flow filtration, hydrophobic interaction and immobilized metal affinity chromatography (IMAC) was employed (data not shown).

### Molecular properties

After chromatographic purification, fractions of highest purity were combined for each variant, resulting in a final pool with an apparent homogeneity of > 98% as judged by SDS/PAGE (data not shown). Recombinant, *Pichia*‐expressed *Am*PDH and all variants showed a broad smear at approximately 90 kDa on SDS/PAGE. Deglycosylation with PNGase F under denaturing conditions resulted in one distinct band of approximately 64 kDa, indicating a degree of glycosylation of approximately 30%, which is in good agreement with previous studies 3, 19. This rather high degree of glycosylation is caused by glycan additions of the high‐mannose type by *P. pastoris* 22 because *Am*PDH purified from its native source *A. meleagris* has a sugar content of approximately 7% 2.

To assess the overall fold of the variants, far‐UV electronic circular dichroism (ECD) spectra were recorded and compared with wild‐type recombinant *Am*PDH. All proteins showed identical far‐UV ECD spectra (data not shown), indicating that they have very similar secondary structure composition 23. Consequently, the mutations introduced did not affect proper protein folding. Distinct minima at 208 and 222 nm indicate a dominating α‐helical content 23, 24, which agrees well with secondary structure elements of the crystal structure of PDH 13.

**Figure 2:**
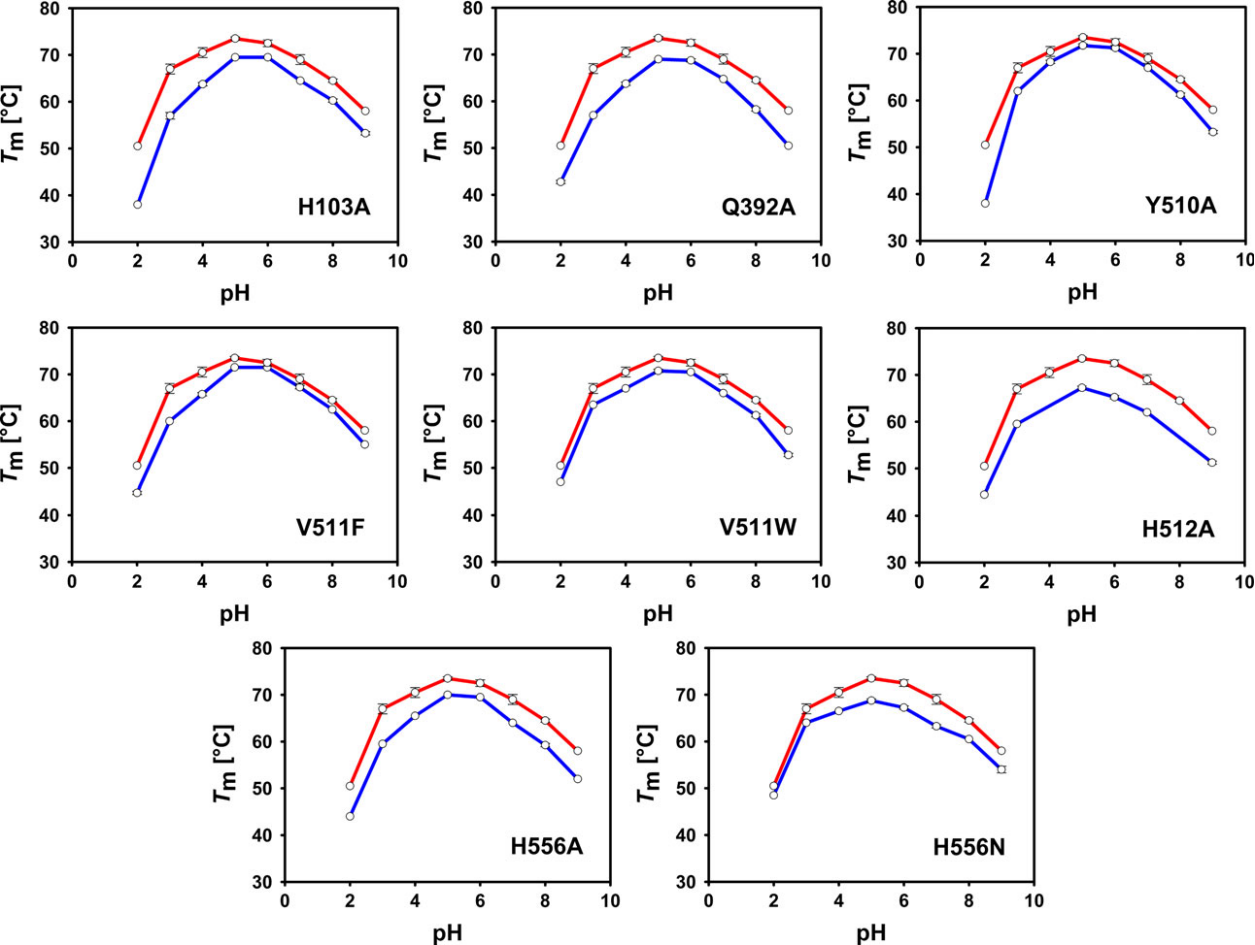
Melting temperatures *T*_m_ obtained from *Thermo* FAD experiments using 65 μm *Am* PDH and its variants in 40 mm Britton‐Robinson buffer (pH 2–9). The upper red line represents the *T*_m_ value of wild‐type recombinant *Am* PDH and the lower blue line is the variant indicated. Because of limited amounts of H512A available, data could only be recorded for pH 2, 3, 5, 6, 7 and 9. Data shown are the mean ± SD value of at least two independent experiments.

To assess the effect of the introduced mutations on thermal stability of the *Am*PDH variants, the unfolding temperature (*T*_m_) was determined by the *Thermo*FAD method 25 in the pH range 2–9 (Fig. 2). All variants are most stable in the pH range 5–6. A *T*_m_ value of 73.5 °C was determined for recombinant wild‐type *Am*PDH. Interestingly, variant H103A has a *T*_m_ of 69.5 °C, which is 5.7 °C higher compared to the H103Y variant studied previously 20. Apparently, the introduction of the bulkier tyrosine side chain at this position destabilizes the protein more significantly. The largest decrease in *T*_m_ for all studied variants was observed for H512A (*T*_m_ = 67.0 °C), which is 6.5 °C lower than the value for wild‐type recombinant *Am*PDH. This suggests that His512 is important in maintaining the active‐site architecture and the overall fold. *T*_m_ values ranging from 68.8 to 71.8 °C were determined for the other variants, indicating that the amino acid replacements chosen have no major effect on thermal stability and do not destabilize any of the variants to an extent that complicates further studies.

**Figure 3:**
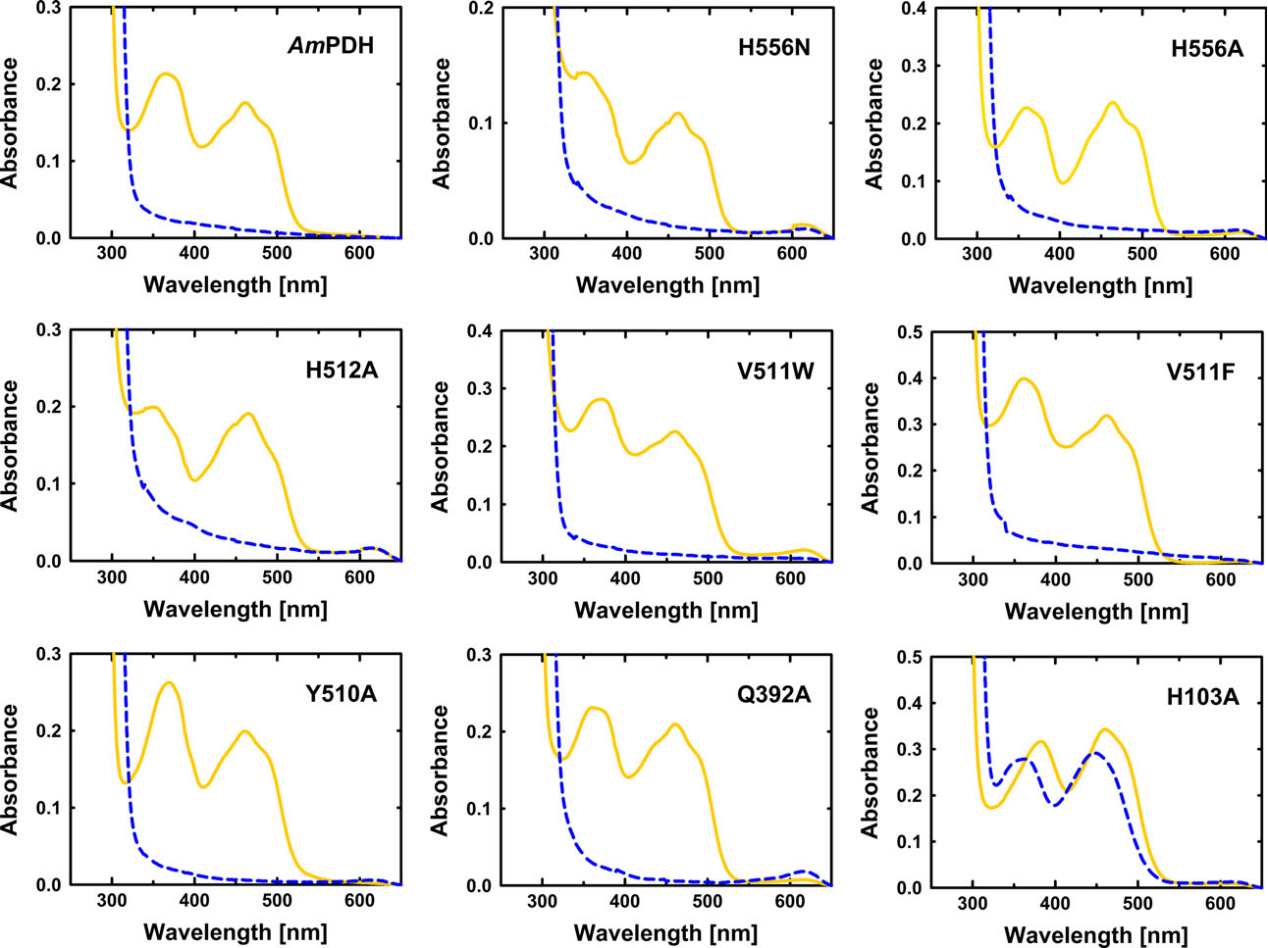
TCA/acetone precipitation of pyranose dehydrogenase from *A. meleagris* and its variants. Initially, UV‐visible spectra of fully oxidized enzymes were recorded (solid line in yellow). After adding 10% (v/v) TCA and 40% (v/v) acetone to the double‐concentrated protein solution and incubation for 10 min on ice, the UV‐visible spectra of the supernatant were recorded again (dashed lines in blue).

Studies on the related enzyme *Tm*POx showed that active‐site variants may contain mixed populations of (non)covalently bound FAD 26, 27. This was also found for amino acid replacements not involved in the covalent linkage as a result from a decrease of the overall positive charge around the flavin N1 region. Consequently, covalent FAD attachment was tested for all generated variants using protein precipitation with 10% trichloroacetic acid (TCA) and 40% acetone. Spectra of the protein solution before and after precipitation were recorded (Fig. 3). For variant H103A, lacking the covalent linkage to FAD, 89% of the protein released the FAD cofactor into the supernatant after precipitation. For *Am*PDH and all other variants, < 4% FAD was found in the supernatant, indicating covalently bound FAD cofactor. The UV‐visible spectra of the oxidized, nonprecipitated enzyme solutions (Fig. 3, black lines) show a hypsochromic (blue) shift of the flavin peak at approximately 370 nm for all variants with a covalently attached FAD. This shift indicates the presence of 8α‐substituted flavin 28, 29, which, in the case of PDH, is an 8α‐*N* ^3^‐histidyl‐FAD 13, 30. Free FAD has a characteristic absorption peak at 450 nm 29, which is red‐shifted in *Am*PDH and all variants. The bathochromic shift of the 450 nm peak upon FAD integration into a protein has been described earlier (e.g. for monomeric sarcosine oxidase) 31. It is caused by a more extended delocalization of the π‐electron system of the aromatic ring system in FAD 32. For H103A, the maximum of this absorption peak blue‐shifts from 460 to 450 nm after protein precipitation (Fig. 3, dashed line), as expected for free FAD.

### Apparent steady‐state kinetics, MD simulations and free energy calculations

Apparent steady‐state kinetic constants were determined for both, the electron donor substrate GLC and for the one‐electron acceptor ferrocenium ion (Fc^+^) (Table 1). For Fc^+^, variants can be classified in two groups: those with *K*_M_ values comparable to the wild‐type (H103A, V511W, H556A) and those with a *K*_M_ value that is at least twice as high (Q392A, Y510A, V511F, H556N). For all variants (except Q392A), the *k*_cat_ values are between 20 and 40 s^−1^ and thus significantly lower than for the wild‐type (135 s^−1^). Because the binding site of Fc^+^, the electron transfer path between the reduced enzyme and the electron acceptor, and the rate‐limiting step in the PDH‐catalysed reaction are unknown, care should be taken not to over interpret the apparent steady‐state kinetic data obtained for Fc^+^, and we therefore refrain from drawing any further mechanistic conclusions.

**Table 1:** Apparent steady‐state kinetic constants of PDH from *A. meleagris* and its variants for the electron donor GLC and the electron acceptor Fc^+^. GLC was probed with the standard Fc^+^ assay (50 mm sodium phosphate buffer, pH 7.5, 30 °C, fixed value of 0.2 mm Fc^+^ as electron acceptor). Fc^+^ was tested in 100 mm borate buffer, pH 8.5, 30 °C. The fixed GLC concentration was adjusted according to the *K*_M_ value of the corresponding variant: 25 mm (ns*Am*PDH, rec*Am*PDH, *Am*PDH), 50 mm (H103A, Q392A, V511W), 250 mm (Y510A) or 500 mm (H556A, H556N, V511F). ND, not determined

Variant	GLC			Fc^+^		
	*K*_M_ (mm)	*k*_cat_ (s^−1^)	*k*_cat_/*K*_M_ (mm ^−1^·s^−1^)	*K*_M_ (mm)	*k*_cat_ (s^−1^)	*k*_cat_/*K*_M_ (mm ^−1^·s^−1^)
ns*Am*PDH^c^	0.82 ± 0.03	45.9 ± 0.3	57.5	0.13 ± 0.03	104 ± 8	802
rec*Am*PDH^a^	0.69 ± 0.09	37.8 ± 1.1	54.8	0.16 ± 0.04	130 ± 11	812
*Am*PDH^b^	0.82 ± 0.01	42.2 ± 0.2	51.5	0.17 ± 0.02	135 ± 7	776
H556N	97.5 ± 4.1	6.40 ± 0.08	0.07	0.47 ± 0.04	22.5 ± 1.3	47.8
H556A	90.0 ± 3.5	12.8 ± 0.2	0.14	0.17 ± 0.01	20.4 ± 0.5	124
H512A	6.19 ± 1.24	0.0010 ± 0.0001	0.0002	ND	ND	ND
V511W	3.73 ± 0.17	18.6 ± 0.2	5.00	0.11 ± 0.01	38.0 ± 1.4	352
V511F	106 ± 5	19.9 ± 0.3	0.19	0.39 ± 0.03	26.4 ± 1.4	66.8
Y510A	60.7 ± 2.0	16.0 ± 0.2	0.26	0.49 ± 0.03	28.8 ± 1.3	59.2
Q392A	6.15 ± 0.21	24.5 ± 0.2	3.98	0.30 ± 0.02	111 ± 4	372
H103A	2.73 ± 0.11	15.6 ± 0.1	5.70	0.16 ± 0.01	34.5 ± 1.3	222

a *Am*PDH produced in the natural source *A. meleagris* 2.

b *Am*PDH recombinantly produced in *P. pastoris* 3.

c *Am*PDH with a His_6_‐tag, recombinantly produced in *P. pastoris* (present study).

Studies employing MD simulations 17 and semi‐rational protein engineering 19 suggested that His512 and His556 can both act as catalytic base during GLC oxidation. H512A shows an approximately 10‐fold increase in *K*_M_ (*K*_M,Glc_ of 6.19 mm) and a 41 200‐fold lower *k*_cat_ (*k*_cat,Glc_ of 0.001 s^−1^) compared to recombinant wild‐type *Am*PDH, indicating its functional importance. These values are in agreement with studies on the H548N variant of *Tm*POx, where His548 corresponds to His512 in PDH. For H548N, *K*_M,Glc_ changed from 0.72 to 1.10 mm, whereas *k*_cat,Glc_ was reduced 46 000‐fold compared to wild‐type *Tm*POx 7. In more detailed studies, His548 in *Tm*POx was identified as the general base abstracting the 2‐hydroxyl proton of GLC and initiating the hydride transfer from the substrate to the flavin 27. For *Am*PDH variants involving His556, *k*_cat,Glc_ is diminished by a factor of 3 (H556A) or 7 (H556N) compared to *Am*PDH, indicating that the sugar is still efficiently oxidized. When using the catalytic efficiencies measured for the His556 variants and wild‐type *Am*PDH to calculate the difference in transition state energies (∆∆*G*_ES‡_) according to Eqn (1), we obtained values of 16.8 and 14.9 kJ·mol^−1^ for H556N and H556A, respectively, which is consistent with the removal of a hydrogen bond when exchanging His556. These findings imply that only His512 takes on the role as the general base in *Am*PDH abstracting a proton from a sugar substrate, whereas His556 appears to be mainly involved in substrate interaction. Consequently, the substrate specificity of *Am*PDH cannot be explained by the presence of two active‐site histidines that can both act as a general base but must be attributed to other factors, such as a diverse hydrogen‐bonding pattern and the dominance of relatively unspecific interactions (e.g. van der Waals) for substrate binding in different poses as observed before 17. This is in agreement with previous studies on the glucose–methanol–choline family member aryl‐alcohol oxidase (PDB code: 3FIM) where active‐site histidines His502 (corresponding to His512 in *Am*PDH) and His546 (corresponding to His556 in *Am*PDH) were shown to have identical roles as proposed for *Am*PDH; namely, proton abstraction and substrate interaction, respectively 4. For glucose 1‐oxidase, similar observations were obtained by experimental and computational studies for active‐site histidines His516 (corresponding to His512 in *Am*PDH) and His559 (corresponding to His556 in *Am*PDH) 33.

Compared to wild‐type recombinant *Am*PDH, the lowest increase (3.3‐fold) in *K*_M,Glc_ was observed for variant H103A, which lacks the covalent bond to FAD, and a reduction in *k*_cat,Glc_ by a factor of only 2.7, resulting in the highest catalytic efficiency obtained for any variant (*k*_cat_/*K*_M_ = 5.7 mm ^−1^·s^−1^). Disrupting the covalent FAD linkage in H103A causes a decrease in flavin oxidative power, which can explain this decrease in the turnover number for GLC from 42.2 to 15.6 s^−1^. The variants V511F and V511W were selected based on an *in silico* study 17, which showed that the backbone atoms of Val511 are important for substrate binding through hydrogen bonds (Fig. 1), and that abolishing these bonds might affect the site of GLC oxidation. In the present study, we wanted to corroborate this hypothesis by introducing Phe and Trp at position 511, with both having bulky side chains that potentially disrupt the interaction between substrate and the backbone. For variant V511F, this worked well, as indicated by the difference in transition state energies ∆∆*G*_ES‡_ for wild‐type *Am*PDH and V511F, for which we calculated a value 14.1 kJ·mol^−1^, which is consistent with the loss of a hydrogen bond. For variant V511W, a *k*_cat_ of 18.6 s^−1^ shows that GLC turnover is not dramatically decreased for this variant. Surprisingly, the difference in transition state energies ∆∆*G*_ES‡_ for this side chain exchange was only 5.88 kJ·mol^−1^, which is counterintuitive because more interference would be expected in the substrate interaction by the bulkier Trp side chain. This result, however, could be explained by MD simulations. The N‐atom of the Trp511 side chain forms two prominent hydrogen bonds with the backbone O‐atom of Asp90 (33.8%) and Pro92 (41.5%), cumulating in an average value of 0.75 observed hydrogen bonds. This hydrogen bonds causes the Trp511 side chain to flip out of the active site and, apparently, it does not interfere significantly with the interaction of the main chain carbonyl oxygen and glucose. Tyr510 was suggested to form the floor of the sugar‐binding pocket in PDH, and to bind a sugar substrate via a stacking interaction. Removal of the aromatic ring as in Y510A resulted in a difference in transition state energies (∆∆*G*_ES‡_) of 13.3 kJ·mol^−1^ compared to wild‐type *Am*PDH, which agrees well to quantum mechanical calculations for the interaction of an aromatic ring with a monosaccharide 34.

To further support experimental results with MD simulations, relative binding free energies (ΔΔ*G*_bind_) for glucose were approximated from the experimentally obtained apparent *K*_M_ values $\left(\Delta \Delta G_{\mathrm{bind}}^{\exp}\right)$ according to Eqn (2), and calculated from MD simulations $\left(\Delta \Delta G_{\mathrm{bind}}^{\mathrm{sim}}\right)$ according to an empirical free‐energy method (Eqn (3)) for wild‐type *Am*PDH and selected variants. Because we mainly wanted to investigate the effects on GLC turnover, only variants in which the exchanged residues are expected to interact directly with GLC were studied by MD simulations. The best match between $\Delta \Delta G_{\mathrm{bind}}^{\exp}$ and $\Delta \Delta G_{\mathrm{bind}}^{\mathrm{sim}}$ was obtained for the parameter set of α = 0.503 and β = 0 in Eqn (3), which resulted in a root mean square error of only 3.9 kJ·mol^−1^. This is in line with previous studies on promiscuous enzymes that identified van der Waals interactions as the main driving force for substrate binding 17, 35, 36, which is reflected in the present study by neglecting the electrostatic β‐term and using the van der Waals dependent α‐term only 36.

The difference between approximated $\Delta \Delta G_{\mathrm{bind}}^{\exp}$ and $\Delta \Delta G_{\mathrm{bind}}^{\mathrm{sim}}$ values is below the thermal noise *k*_B_T (2.5 kJ·mol^−1^) for Q392A, V511W, H556A and H556N (Table 2). For variants Y510A, V511F and H512A, the differences in $\Delta \Delta G_{\mathrm{bind}}^{\exp}$ and $\Delta \Delta G_{\mathrm{bind}}^{\mathrm{sim}}$ ranged from 4 to 7 kJ·mol^−1^, which is slightly higher than the chemical accuracy 37, 38 of 4 kJ·mol^−1^. For Y510A, both $\Delta \Delta G_{\mathrm{bind}}^{\exp}$ and $\Delta \Delta G_{\mathrm{bind}}^{\mathrm{sim}}$ are very high, indicating a qualitative agreement. According to $\Delta \Delta G_{\mathrm{bind}}^{\exp}$ for V511F, the Phe side chain in this variant appears to cause steric clashes that interfere with tight substrate binding, resulting in its faster dissociation. To simulate the unbinding, much longer simulation time scales would be necessary, which is beyond the scope of the present study. For variant H512A, which lacks the catalytic His512, the difference between $\Delta \Delta G_{\mathrm{bind}}^{\exp}$ and $\Delta \Delta G_{\mathrm{bind}}^{\mathrm{sim}}$ is possibly caused by a different reaction mechanism compared to *Am*PDH. The very low but measurable residual activity of H512A could probably be derived from rarely occurring solvent‐mediated proton abstraction of the GLC hydroxyl group attached to C2 or C3. This would allow for a hydride transfer to occur between the GLC hydrogen atom attached to C2 or C3 and the flavin N5‐atom. $\Delta \Delta G_{\mathrm{bind}}^{\exp}$ would hence refer to a different species or catalytic binding pose. Therefore, we refrain from drawing any further conclusions based on the conducted MD simulations.

**Table 2:** Relative binding free energies of PDH from *A. meleagris* for glucose. Relative binding free energies (ΔΔ*G*_bind_) for glucose approximated from experimental apparent *K*_M_ values $\left(\Delta \Delta G_{\mathrm{bind}}^{\exp}\right)$ according to Eqn (2), and ΔΔ*G*_bind_ values calculated from MD simulations $\left(\Delta \Delta G_{\mathrm{bind}}^{\mathrm{sim}}\right)$ according to an empirical free‐energy method (Eqn (3)). $\Delta \Delta G_{\mathrm{bind}}^{\mathrm{sim}}$ was calculated with α = 0.503 and β = 0

Variant	ΔΔ*G*_bind_ (kJ·mol^−1^)	
	$\Delta \Delta G_{\mathrm{bind}}^{\exp}$	$\Delta \Delta G_{\mathrm{bind}}^{\mathrm{sim}}$
Q392A	5.1	5.6
Y510A	10.8	15.8
V511F	12.2	5.2
V511W	3.8	2.9
H512A	5.1	9.8
H556A	11.8	9.8
H556N	12.0	9.7

### Product analyses via GC‐MS verify the predictions from MD simulations for the glucose oxidation mode

**Figure 4:**
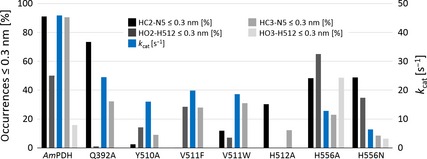
Distance distributions obtained from MD simulations between the GLC hydrogen atom attached to C2 or C3 (HC2 and HC3) and the flavin N5‐atom and the GLC hydroxyl hydrogen atom attached to C2 or C3 (HO2 and HO3) and the His512 Nδ‐ or Nε‐atom, given in occurrences ≤ 0.3 nm [%]. The distance distributions are compared with the experimentally derived *k*_cat_ values (s^−1^) for GLC in blue bars (Table 1). Only variants in which the exchanged amino acid directly interacts with GLC were considered in MD simulations.

**Figure 5:**
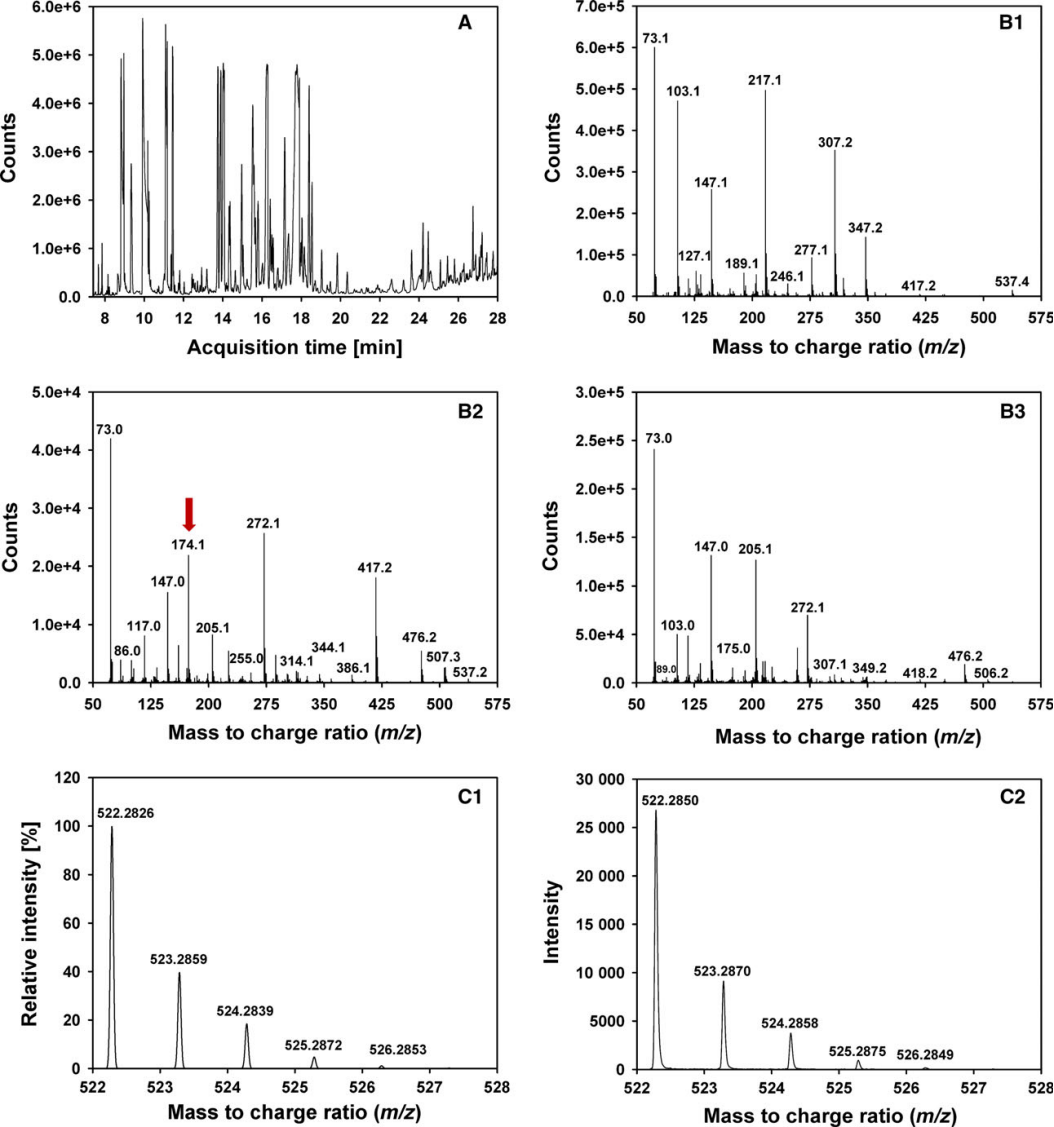
GC‐MS measurements of the products of glucose oxidation catalysed by *Am* PDH. (A) Total ion chromatogram of the *Am* PDH reaction products after 120 min on the HP1MS column with EI‐MS detection. EI‐MS of two‐step derivatizations of 2‐keto‐d‐glucose (B1), 3‐keto‐d‐glucose (B2) and 2,3‐diketo‐d‐glucose (B3). The red arrow in (B2) indicates the peak that was used to differentiate between 2‐keto‐d‐glucose and 3‐keto‐d‐glucose. Isotopologue fraction of the protonated molecular ion of ethoxymation‐trimethylsilylation derivatization products of 2,3‐diketo‐d‐glucose: theoretical distribution (C1) and experimental distribution (C2).

We measured the distances between: (i) the GLC hydrogen atom attached to C2 or C3 (HC2 and HC3) and the flavin N5‐atom and (ii) the hydrogen atom of the hydroxyl group of GLC attached to C2 or C3 (HO2 and HO3) and the His512 Nδ‐ or Nε‐atom in MD simulations. These distances were successfully employed to reproduce and predict C2 and C3 oxidation of various monosaccharides by *Am*PDH in recent studies 17, 18, in which a 0.3 nm cut‐off was used to discriminate between reactive (≤ 0.3 nm) and nonreactive distances (i.e. enabling proton abstraction and hydride transfer). A qualitative agreement between the occurrences of reactive distances of less than 0.3 nm and the experimentally determined *k*_cat,Glc_ values is observed (Fig. 4). When multiplying the probability of occurrences of distances of ≤ 0.3 nm for HC‐N5 with those for HO‐H512 (for C2 or C3, respectively), the product always yields a higher value for C2 compared to C3, indicating that C2 is more likely to be positioned for proton abstraction and hydride transfer than C3. This suggests that oxidation at C2 is preferred over C3. To confirm this hypothesis, GC‐MS measurements were conducted to quantify GLC and its oxidized reaction products. We used a novel approach of two‐step derivatization (ethoxylation and trimethylsilylation), capillary gas chromatography and electron ionization mass spectrometry (GC‐EI‐MS) to quantify GLC and its (di‐)keto reaction products 39, 40. Because authentic standards for 3‐keto‐GLC and 2,3‐diketo‐GLC are not commercially available, capillary gas chromatography combined with chemical ionization time of flight mass spectrometry (GC‐CI‐QToFMS) was employed to confirm their identity via accurate mass, isotopologue pattern and modified retention indices (a detailed description of the GC‐MS measurements is provided in the Materials and methods, as well as in Fig. 5 and Table 3). A total ion chromatogram of a sample analysed by GC‐EI‐MS is presented in Fig. 5A. The standard electron ionization (70 eV) mass spectra of the two‐step derivatization product of 2‐keto‐GLC was confirmed via standard EI mass spectra with low resolution, which gave an *m*/*z* of 552.4 (M+, < 1%), 537.4 ([M‐CH_3_]+, 5%), 417.2 (5%), 347.2 (18%), 307.2 (40%), 277.1 (12%), 246.1 (5%), 217.1 (60%), 147.1 (35%), 103.1 (65%) and 73.1 (100%, trimethylsilylium ion). Mass spectra of the two‐step derivatization product of 2‐keto‐GLC were successfully cross‐checked with published data. In addition, the fragmentation pathway of the two‐step derivatization products of 2‐keto‐GLC and 3‐keto‐GLC was predicted with mass frontier, version 7.0SP1 (Thermo Scientific, Waltham, MA, USA). For 3‐keto‐GLC, the mass spectrum of the two‐step derivatization products was similar to 2‐keto‐GLC, except for one fragment derived from 3‐keto‐GLC with an *m*/*z* of 174.1, which differed significantly between two isomers. With this fragment and the protonated ion in methane, chemical ionization spectra were used to screen for this compound in the enzyme reactions. However, a high degree of fragmentation during electron ionization and unspecific fragments in standard electron ionization were observed, preventing the distinction between 2‐keto‐GLC and 3‐keto‐GLC by only EI or CI mass spectra. Because of the lack of commercially available standards for 3‐keto‐GLC and 2,3‐diketo‐GLC, the discrimination between the different keto‐GLC derivatives was only possible by combining chemical ionization mass spectra and electron ionization mass spectra with the fragmentation pathway. The molecular formula generation was calculated via the compound identification function in masshunter (Agilent Technologies, Santa Clara, CA, USA). Elements (C, N, O and Si) and the number of atoms were limited in accordance with derivatization products of mono‐ and diketo‐GLC.

**Table 3:** Retention indices and mass accuracy of derivatization products of GLC, mono‐ and diketo‐GLC

No	Analytes	Chemical formula of quasi‐molecular ion	Retention index	Theoretical mass (Da)	Measured mass (Da)	Mass accuracy (p.p.m.)
1	2‐keto‐GLC	[C_22_H_53_N_2_O_6_Si_4_]^+^	2162	553.2975	553.2981	1.0
2	3‐keto‐GLC	[C_22_H_53_N_2_O_6_Si_4_]^+^	2192	553.2975	553.3050	13.6
3	2,3‐diketo‐GLC	[C_21_H_48_N_3_O_6_Si_3_]^+^	2157	522.2845	522.2850	0.9
4	GLC	[C_23_H_58_O_6_Si_5_]^+^	2188	584.3105	584.3092	2.2

The chemical formula of the derivatization product of 2‐keto‐GLC and 3‐keto‐GLC is C_22_H_52_N_2_O_6_Si_4_ with an accurate mass of 552.2897 Da, and C_21_H_47_N_3_O_6_Si_3_ for 2,3‐diketo‐GLC with an accurate mass of 521.2767 Da. The *m*/*z* of the compounds was increased by 1.0080 Da after ionization by methane as chemical reagent gas. The theoretical isotopologue fractions of derivatization product ions of mono‐ and diketo‐GLC were calculated via envipat (http://www.envipat.eawag.ch/index.php). High‐resolution spectra of exthoxymation‐trimethylsilylation of mono‐ and diketo‐GLC employing methane gas ionization are shown in Fig. 5B. For the investigated mono‐ and diketo‐GLC, the mass accuracy of the protonated molecular ion was below 15 p.p.m. and the experimental isotopologue fractions were in agreement with the theoretical mass (Table 3). Identification of 2,3‐diketo‐GLC was accomplished by its retention time during chromatography. In addition, the molecular formula generation function in qualitative masshunter with atoms filter C, H, N, O and Si was set to 19‐23, 47‐55, 2‐4, 5‐8 and 2‐5, respectively. The comparison of selected compounds with the molecular formula generation was carried out not only by *m*/*z* and isotopologue fraction, but also according to the spacer between the isotopic patterns. The match scores were 93.9 and 96.4 for mass and isotopologue fraction distribution, respectively. Figure 5C shows the theoretical and experimental isotopologue fractions for 2,3‐diketo‐GLC.

The concentrations and relative ratios of GLC and its (di‐)keto reaction products obtained from GC‐MS measurements for *Am*PDH and its variants after 3, 8 and 120 min of reaction time are listed in Table 4. Only the reaction products of variant H512A could not be identified because of its extremely slow GLC turnover (Table 1). These results match the predictions from MD simulations very well, showing that C2 oxidation is indeed preferred over C3 oxidation because only very little or no 3‐keto‐GLC was detected, whereas there were significant amounts of 2‐keto‐GLC and 2,3‐diketo‐GLC for all tested variants. This indicates that 2‐oxidation is significantly faster than 3‐oxidation, for both GLC and a mono‐oxidized intermediate.

**Table 4:** GC‐MS analysis of GLC and its reaction products 2‐keto‐GLC, 3‐keto‐GLC and 2,3‐diketo‐GLC. The reaction mixture contained 1 mL of 10 mm potassium phosphate buffer (pH 7.0), 1 U of *Am*PDH or its respective variant, 45 mm 1,4‐benzoquinone as electron acceptor and, initially, 16.7 mm GLC. Samples (200 μL) were taken after 3, 8 and 120 min of reaction time, respectively. After deproteinization through a 10 kDa ultrafiltration membrane, the filtrate was used for subsequent GC‐MS analysis. For GLC and 2‐keto‐GLC, an external calibration was possible with commercially available standards, allowing for a quantitative determination of their concentrations (mm) with a detection limit of 0.2 μm. Because no standards were commercially available for 3‐keto‐GLC and 2,3‐diketo‐GLC, only semiquantitative results (relative concentrations in mm) could be calculated, based on the peak‐area‐ratios of the respective analyte to 2‐keto‐GLC at a given mass to charge ratio. To obtain the ‘Ratio’, the percentage of each compound with respect to the sum of (relative) concentrations was calculated for each variant

Variant	Reaction time (min)	Concentration (mm)		Relative concentration (mm)		Ratio (%)			
		GLC	2‐keto‐GLC	3‐keto‐GLC	2,3‐diketo‐GLC	GLC	2‐keto‐GLC	3‐keto‐GLC	2,3‐diketo‐GLC
*Am*PDH	3	2.69	1.72	0.000	0.061	60.2	38.5	0.0	1.4
	8	2.43	1.69	0.001	0.273	55.4	38.4	0.0	6.2
	120	0.43	1.33	0.026	1.271	14.0	43.6	0.9	41.6
H103A	3	3.42	0.597	0.000	0.100	83.1	14.5	0.0	2.4
	8	2.91	1.65	0.004	0.342	59.3	33.7	0.1	7.0
	120	1.62	1.49	0.024	1.108	38.2	35.1	0.6	26.1
Q392A	3	3.07	1.50	0.002	0.186	64.5	31.6	0.0	3.9
	8	2.63	1.87	0.002	0.724	50.3	35.8	0.0	13.9
	120	1.04	1.49	0.032	1.540	25.4	36.2	0.8	37.6
Y510A	3	3.35	0.126	0.000	0.002	96.3	3.6	0.0	0.1
	8	3.40	0.386	0.000	0.034	89.0	10.1	0.0	0.9
	120	2.73	1.59	0.010	0.401	57.7	33.6	0.2	8.5
V511F	3	3.62	0.311	0.001	0.005	91.9	7.9	0.0	0.1
	8	3.60	1.09	0.001	0.030	76.3	23.1	0.0	0.6
	120	2.42	2.46	0.025	0.387	45.7	46.5	0.5	7.3
V511W	3	3.34	1.70	0.001	0.058	65.5	33.4	0.0	1.1
	8	3.04	2.46	0.010	0.331	52.0	42.1	0.2	5.7
	120	0.275	1.84	0.150	1.564	7.2	48.0	3.9	40.9
H556A	3	3.86	0.293	0.001	0.018	92.5	7.0	0.0	0.4
	8	3.46	0.811	0.000	0.075	79.6	18.6	0.0	1.7
	120	3.54	2.80	0.001	0.466	52.0	41.2	0.0	6.8
H556N	3	3.92	0.090	0.000	0.003	97.7	2.2	0.0	0.1
	8	3.66	0.187	0.000	0.012	94.8	4.9	0.0	0.3
	120	3.45	1.17	0.001	0.110	72.9	24.7	0.0	2.3

Based on these results, we propose a scheme for the two‐step oxidation of GLC by *Am*PDH that is also valid for all tested variants. This scheme is very similar to the one reported earlier by Volc *et al*. 41 for PDH from *Agaricus bisporus* (*Ab*PDH). The main difference between *Ab*PDH and *Am*PDH is that *Am*PDH almost exclusively double‐oxidizes GLC via 2‐keto‐GLC, whereas *Ab*PDH performs this reaction via both 2‐keto‐GLC and 3‐keto‐GLC, with the latter being favoured to some extent, to yield the final product 2,3‐diketo‐GLC.

In a previous *in silico* study, mutations at Gln392, Tyr510 and Val511 in *Am*PDH were suggested to modify the preferred GLC oxidation site because these amino acids are involved in prominent hydrogen bonds with the substrate bound in a position relevant for either C2 or C3 oxidation 17. Interestingly, neither explicit simulations of the mutants, nor the GC‐MS analysis after glucose turnover indicated an altered preference for C2 or C3 oxidation compared to wild‐type *Am*PDH. Consequently, substrate binding in an orientation specific for either C2 or C3 oxidation cannot be attributed to one specific active‐site residue in *Am*PDH, although an interplay between several residues appears to be critical for this.

### C2 and C3 oxidation of various sugar substrates proceed with comparable rates

**Figure 6:**
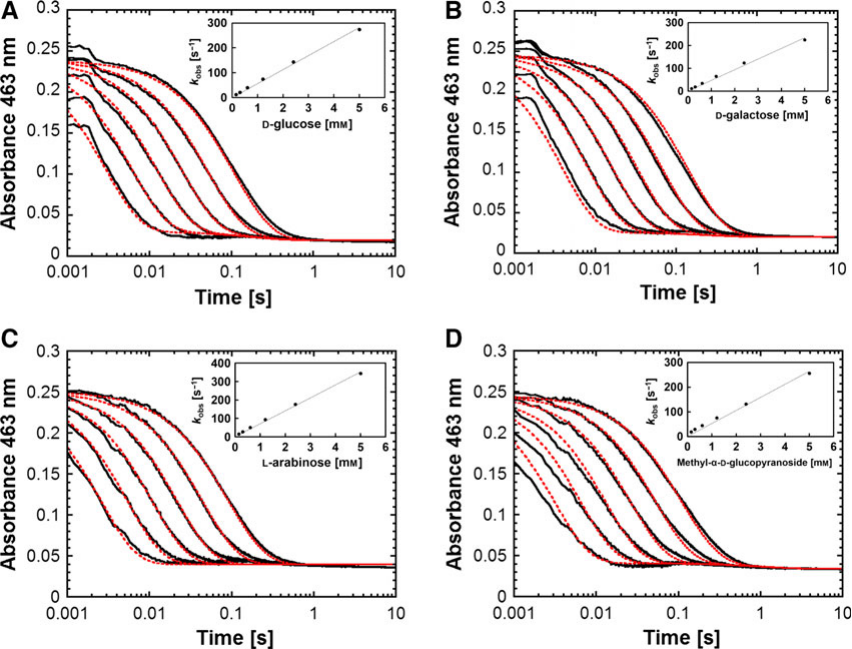
Reductive half‐reaction of pyranose dehydrogenase from *A. meleagris* followed by stopped‐flow spectroscopy after mixing with sugar substrates (A) GLC, (B) GAL, (C) ARA and (D) MGP. A solution of fully oxidized enzyme (30 μm) was mixed with buffer containing various sugar concentrations (0.15, 0.3, 0.6, 1.2, 2.4 and 5 mm in 50 mm potassium phosphate buffer, pH 7.0) at 4 °C. All given concentrations are reported after mixing. The reaction was monitored at 463 nm. The kinetic traces from right to left correspond to increasing sugar concentrations. To evaluate the experimental traces (black), a double‐exponential fit was used. For all sugars, the first phase *k*_obs(1)_ was the FAD reduction with a large absorbance decrease at 463 nm, which was used to calculate *k*_app,4 °C_. The experimental traces were simulated with berkeley madonna, version 8.3.14, according to Kinetic Model 1_red_ (red traces) with the parameter sets listed in Table 5. The inset shows the plot of the pseudo first‐order rate constants *k*_obs(1)_ versus the concentrations of the respective sugar.

The reductive half reaction (FAD → FADH_2_) of *Am*PDH was studied in detail with sugar substrates that are specifically and exclusively oxidized at either C2 or C3 to investigate whether *Am*PDH shows a preferred mode of sugar oxidation (i.e. whether the rate constants are significantly different for C2 or C3 oxidation). As reported previously, *Am*PDH is reduced rapidly by GLC at 30 °C, with an apparent bimolecular rate constant (*k*_app,30 °C_) of 1.1 ± 0.03 × 10^5^ m ^−1^·s^−1^ 20. Consequently, all stopped‐flow experiments in the present study were conducted at 4 °C to slow down the reaction and capture as much kinetic information as possible. All kinetic traces obtained (Fig. 6) were simulated with parameters listed in Table 5 according to Kinetic Model 1_red_:

**Table 5:** Parameter set for simulating stopped‐flow traces with berkeley madonna, version 8.3.14. The reductive half‐reaction recorded for different electron donors (sugars) was simulated in agreement with Kinetic Model 1_red_. The oxidative half‐reaction for the electron acceptor BQ was simulated according to Kinetic Model 1_ox_. Simulated traces of the reductive and oxidative half‐reactions for *Am*PDH are shown in red dashed lines in Figs 6 and 7, respectively

Variant	Substrate	*k*_1_ (m ^−1^·s^−1^)	*k*_2_ (m ^−1^·s^−1^)	ε_A_ (m ^−1^·cm^−1^)	ε_B_ (m ^−1^·cm^−1^)	ε_C_ (m ^−1^·cm^−1^)
*Am*PDH	GLC	6.9 × 10^4^	5.4	9.0 × 10^3^	1.1 × 10^3^	0.7 × 10^3^
	GAL	5.2 × 10^4^	4.0	9.3 × 10^3^	1.0 × 10^3^	0.8 × 10^3^
	ARA	7.8 × 10^4^	5.0	9.5 × 10^3^	1.5 × 10^3^	1.5 × 10^3^
	MGP	6.5 × 10^4^	1.0	9.3 × 10^3^	1.5 × 10^3^	1.3 × 10^3^
	BQ	8.0 × 10^3^	0.05	1.7 × 10^3^	1.3 × 10^4^	9.2 × 10^3^
H556A	GLC	1.0 × 10^2^	8.0 × 10^1^	1.3 × 10^4^	9.8 × 10^3^	1.1 × 10^3^
	BQ	3.6 × 10^4^	1.6 × 10^3^	1.2 × 10^3^	2.0 × 10^4^	1.1 × 10^4^

$$E_{\text{ox}}+S\overset{k_{1}}{\underset{}{\to }}E_{\text{red}}:P\overset{k_{2}}{\underset{}{\to }}E_{\text{red}}+P\;\left(\text{Kinetic Model}\;1_{\text{red}}\right)$$

According to previously published data 1, 16, d‐galactose (GAL) and l‐arabinose (ARA) are exclusively oxidized at C2, whereas methyl‐α‐d‐glucopyranoside (MGP) is only oxidized at C3, with no double oxidation occurring. *Am*PDH oxidizes GLC at both C2 and C3 but C3 oxidation (also of mono‐oxidized 2‐keto‐GLC) is very slow and takes minutes to hours 13 (Table 4). Therefore, GLC can be treated as C2‐oxidized substrate in the time course of stopped‐flow experiments that spans just a few seconds. The reactions of *Am*PDH for GLC, GAL, ARA and MGP monitored at 463 nm were biphasic. Their time traces were fitted by a double‐exponential function to obtain the respective *k*_obs_ values at distinct sugar concentrations. The first phase *k*_obs(1)_ corresponded to FAD reduction with a large absorbance decrease at 463 nm. It was used for calculating *k*_app,4 °C_ (Table 6). The second phase *k*_obs(2)_ showed a small decrease in absorbance at the same wavelength and was independent of the GLC concentration (data not shown). The *y*‐intercepts between 7.6 and 15.2 s^−1^ indicate a reversible step that cannot be significant because the magnitude of the *y*‐intercept is rather small compared with the scale of the *y*‐axis for all tested sugars (Fig. 6). Moreover, the simulated traces (Kinetic Model 1_red_) agree well with experimental traces (Fig. 6), indicating the validity of the model employed, which does not consider a reversible step. For all sugars, the obtained *k*_app,4 °C_ values were similar and in a range of 4.4 to 6.8 × 10^4^ m ^−1^·s^−1^ (Table 6). The pentose sugar ARA showed the highest *k*_app,4 °C_, which can possibly be attributed to its smaller size compared to the other tested sugars (hexoses) because this could ultimately facilitate its diffusion to and from the active site.

**Table 6:** Stopped‐flow spectroscopy for the reductive half‐reaction of *Am*PDH with GLC, GAL, ARA and MGP and the oxidative half‐reaction with BQ. For variant H556A, only GLC and BQ were used. Experiments were performed in 50 mm potassium phosphate buffer (pH 7.0) and 4 °C. Stopped‐flow traces were simulated with berkeley madonna, version 8.3.14, according to Kinetic Model 1_red_ or Kinetic Model 1_ox_. Employed enzyme and substrate concentrations, monitored wavelengths and experimental and simulated traces are provided in Figs 6 and 7. Additional details on GC‐MS measurements to determine the GLC oxidation sites for H556A are provided in Table 4. *y*‐axis intercepts for *Am*PDH were 7.61 (GLC), 9.11 (GAL), 9.06 (ARA), 15.2 (MGP) and 0.92 (BQ). *y*‐axis intercepts for H556A were zero for GLC and BQ

Variant	Substrate	Oxidation site	Reference for oxidation site	Apparent bimolecular rate constant *k*_app_ (m ^−1^·s^−1^)	
				Experiment	Simulation
*Am*PDH	GLC	C2/3^a^	11, 16	5.4 ± 0.11 × 10^4^	6.9 × 10^4^
	GAL	C2	16	4.4 ± 0.15 × 10^4^	5.2 × 10^4^
	ARA			6.8 ± 0.10 × 10^4^	7.8 × 10^4^
	MGP	C3	12	4.8 ± 0.05 × 10^4^	6.5 × 10^4^
	BQ	–	–	6.6 ± 0.17 × 10^3^	8.0 × 10^3^
H556A	GLC	C2/3^a^	Present study	7.3 ± 0.24 × 10^1^	8.0 × 10^1^
	BQ	–	–	4.1 ± 0.02 × 10^4^	3.6 × 10^4^

a Predominantly oxidized at C2 and only very slowly at C3 (Table 4).

### Effects of the His → Ala replacement at position 556 on both half reactions

Although His556 does not act as a catalytic base, we found that it is important for sugar substrate binding (see above). To identify the rate‐limiting step in variant H556A and compare the results with recombinant wild‐type *Am*PDH, the transient‐state kinetics of their reductive (FAD → FADH_2_) and oxidative (FADH_2 _→ FAD) half‐reactions were investigated by stopped‐flow spectroscopy, using GLC as electron donor and 1,4‐benzoquinone (BQ) as electron acceptor, respectively. In a previous study, the ferrocenium ion (Fc^+^) was used as electron acceptor, which has an absorbance at 330–350 nm and 625 nm 20. Therefore, Fc^+^ interferes to a greater extent with the flavin absorbance than BQ, which has an absorption maximum at 244 nm, and the corresponding hydroquinone at 222 nm and 296 nm, respectively 42. The stopped‐flow experiments were performed essentially as described above, and the kinetics were recorded at 465 nm for H556A and at 463 nm for *Am*PDH, respectively. For both GLC and BQ, the reactions were biphasic and their time traces were fitted by a double‐exponential function to obtain the *k*_obs,4 °C_ values.

**Figure 7:**
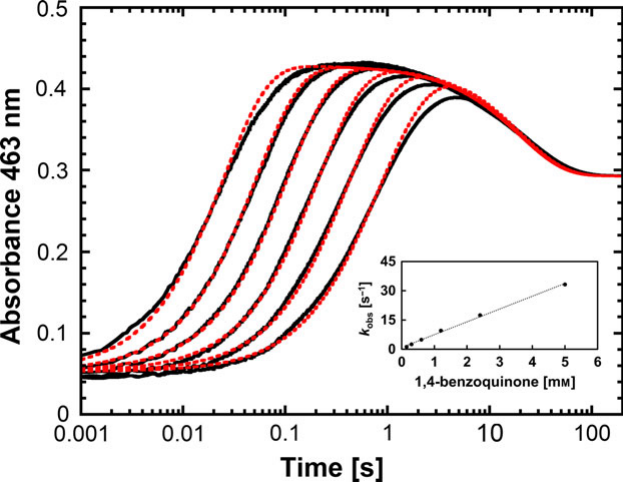
Oxidative half‐reaction of pyranose dehydrogenase from *A. meleagris* followed by stopped‐flow spectroscopy after mixing with BQ. A solution of fully reduced enzyme (30 μm) was mixed with buffer containing various BQ concentrations (0.15, 0.3, 0.6, 1.2, 2.4 and 5 mm in 50 mm potassium phosphate buffer, pH 7.0) at 4 °C under anaerobic conditions. All given concentrations are reported after mixing. The reaction was monitored at 463 nm. The kinetic traces from right to left correspond to increasing BQ concentrations. To evaluate the experimental traces (black), a double‐exponential fit was used. The first phase *k*_obs(1)_ was the FAD oxidation with a large absorbance increase at 463 nm, which was used to calculate *k*_app,4 °C_. The experimental traces were simulated with berkeley madonna, version 8.3.14, according to Kinetic Model 1_ox_ (red traces) with the parameter sets listed in Table 5. The inset shows the plot of the pseudo first‐order rate constants *k*_obs(1)_ versus the BQ concentrations.

As described above, a *k*_app,4 °C_ of 5.4 ± 0.11 × 10^4^ m ^−1^·s^−1^ was obtained for the reductive half‐reaction of *Am*PDH with GLC (Table 6), corresponding to a two‐fold decrease compared to measurements at 30 °C 20. For the oxidative half‐reaction of *Am*PDH with BQ, the first phase *k*_obs(1)_ of the double‐exponential fit corresponds to FAD oxidation with a large absorbance increase at 463 nm (Fig. 7), which was used to calculate *k*_app,4 °C_ (Table 6). The second phase *k*_obs(2)_ of the double‐exponential fit showed a significant absorbance decrease from the intermediate species *E*_ox_:BQH_2_ to *E*_ox_ and was independent of the BQ concentration (data not shown). For the first phase, the *y*‐intercept is very small, indicating a negligible reversible step. Simulations according to Kinetic Model 1_ox:_

$$E_{\text{red}}+\text{BQ}\overset{k_{1}}{\underset{}{\to }}E_{\text{ox}}\;\;:\;{\text{BQH}}_{2}\overset{k_{2}}{\underset{}{\to }}E_{\text{ox}}+{\text{BQH}}_{2}\;\left(\text{Kinetic}\;\text{Model}\;1_{\text{ox}}\right)$$

with parameter sets listed in Table 5 corroborate this observation because the simulated traces agree well with experimental traces (Fig. 7), and the model does not consider a reversible step as well. The second phase of simulated and experimental traces does not overlap as well as the first phase, which might be attributed to the complex quinone reduction process or to polymerizations during side reactions. However, the imperfect fit does not affect the first phase, which was used to calculate *k*_obs(1)_ for *Am*PDH oxidation, and therefore the utilized kinetic model is valid.

For the reductive half‐reaction of H556A with GLC, the first phase *k*_obs(1)_ of the double‐exponential fit only showed a small absorbance decrease and was independent of the GLC concentration (data not shown). The second phase *k*_obs(2)_ of the double‐exponential fit corresponds to FAD reduction with a large absorbance decrease at 465 nm (data not shown), which was used to calculate *k*_app,4 °C_ (Table 6). For the second phase, the *y*‐intercept is close to zero, indicating a negligible reversible step. The *k*_app,4 °C_ for the reductive half‐reaction of variant H556A with GLC dramatically decreased 720‐fold compared to *Am*PDH (Table 6), indicating that the two‐electron transfer to FAD is significantly hampered by this amino acid exchange.

In the oxidative half reaction of H556A with BQ, the first phase *k*_obs(1)_ of the double‐exponential fit corresponds to FAD oxidation with a large absorbance increase at 465 nm, which was used to calculate *k*_app,4 °C_ (Table 6). The second phase *k*_obs(2)_ of the double‐exponential fit only showed a small absorbance increase and was independent of the BQ concentration (data not shown). For the first phase, the *y*‐intercept is again close to zero, which indicates no reversible step. Similar to *Am*PDH, the second phase of simulated and experimental traces for the oxidative half‐reaction in H556A do not perfectly overlap. For variant H556A, the *k*_app,4 °C_ of the biphasic oxidative half‐reaction with BQ was approximately 6.2‐fold higher compared to *Am*PDH (Table 6), which can possibly be attributed to the lower redox potential of H556A (see below) or to a more accessible active‐site in the variant. However, because the binding site of BQ in *Am*PDH and the electron transfer path from *Am*PDH to BQ remain elusive, further studies will be necessary before any final conclusions can be drawn.

The presented pre‐steady‐state experiments clearly demonstrate that the impairment of the reductive half‐reaction of variant H556A is the main reason for its considerably lower catalytic efficiency (Table 1) compared to *Am*PDH. The oxidative half‐reaction of variant H556A is significantly faster compared to *Am*PDH.

### Disruption of the covalent FAD linkage decreases the redox potential

The covalent FAD attachment in flavin‐dependent proteins, and especially the attachment of electron‐withdrawing substituents at position 8, has been associated with the increase in redox potential of the enzymes 30, 43, allowing them to use high‐potential electron acceptors such as molecular oxygen as redox partners. Consequently, a covalently attached FAD was mainly found in oxidases. This makes *Am*PDH a rather unusual example for a dehydrogenase in carrying an FAD that is monocovalently tethered via position 8. Therefore, we probed the effect of the missing covalent FAD linkage in H103A on its redox potential. Furthermore, the decrease in the reductive half‐reaction of variant H556A could potentially be explained by a decrease in the redox potential of this variant, and hence we determined its redox potential as well.

**Figure 8:**
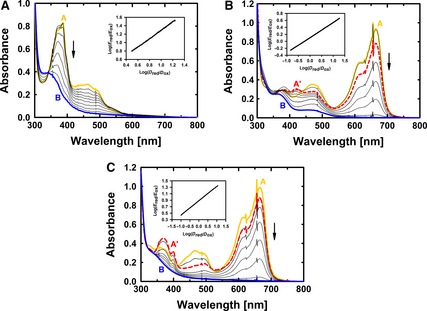
Determination of reduction potentials $\left(E^{o\prime }\right)$ according to Massey's method 70 for pyranose dehydrogenase from *A. meleagris* (A), variant H103A (B) and variant H556A (C). Spectral line A (uppermost solid line in yellow) represents the fully oxidized enzyme and dye, whereas spectral line B (lowest solid line in blue) represents fully reduced enzyme and dye. In (B) and (C), the dashed red line A′ represents the maximum of an occurring semiquinone intermediate with a maximum absorbance at 422 nm (B) or 368 nm (C). The inset in (A) shows the reduction of phenazine methosulfate (dye, ‘D’) monitored at 387 nm and the reduction of *Am* PDH (enzyme, ‘E’) monitored at 463 nm, allowing the calculation of the ratios between reduced and oxidized species (*E*_red_/*E*_ox_ and *D*_red_/*D*_ox_). $E^{o\prime }$ of *Am* PDH was calculated to be +92 ± 3 mV. The inset in (B) shows the reduction of methylene blue (‘D’) monitored at 664 nm and the reduction of variant H103A (‘E’) monitored at 460 nm. $E^{o\prime }$ of variant H103A was calculated to be +63 ± 3 mV. The inset in (C) shows the reduction of methylene blue (*D*) monitored at 664 nm and the reduction of variant H556A (*E*) monitored at 465 nm. $E^{o\prime }$ of variant H556A was calculated to be +84 ± 4 mV. An overview of the obtained data is provided in Table 7.

The reduction of *Am*PDH (Fig. 8A and Table 7) followed a two‐electron reduction process as judged by a slope of 1.0 when plotting log(*E*_red_/*E*_ox_) versus log(*D*_red_/*D*_ox_). The midpoint potential $\left(E^{o\prime }\right)$ for *Am*PDH was +92 ± 3 mV at pH 7.0 and 25 °C. For H103A (Fig. 8B and Table 7), the fully oxidized enzyme was reduced by two one‐electron reduction steps, as indicated by a slope of 0.41 when plotting log(*E*_red_/*E*_ox_) versus log(*D*_red_/*D*_ox_). Thus, the reduction of H103A to the fully reduced enzyme proceeds via a putative semiquinone intermediate, which could however not be observed in anaerobic redox titrations with dithionite (data not shown). The $E^{o\prime }$ of the overall reduction was calculated according to Eqns (5), (6) (see also Materials and methods). The observed semiquinone intermediate for variant H103A had an absorption maximum at 422 nm (dashed line in Fig. 8B). This does not perfectly fit to the characteristic absorbance of the anionic semiquinone radical at approximately 380 nm and 400 nm 29. However, it is known that the microenvironment around the flavin (e.g. the polypeptide matrix, pH, ligands) can significantly affect the occurrence of semiquinone radicals 29 and can cause bathochromic shifts of, for example, the 450 nm flavin absorption band 32. Consequently, the red‐shifted semiquinone absorbance in variant H103A can possibly be attributed to similar factors. For variant H103A, $E^{o\prime }$ was +63 ± 3 mV at pH 7.0 and 25 °C (Table 7). Therefore, disruption of the covalent FAD linkage decreases the redox potential by 29 mV.

**Table 7:** Redox potential values $\left(E^{o\prime }\right)$ for PDH from *A. meleagris* and selected variants. The corresponding reduction spectra are plotted in Fig. 8. A slope close to 1.0 indicates a two‐electron reduction step, which explains why no one‐electron reduction steps $\left(E_{1}^{o\prime }\text{and}E_{2}^{o\prime }\right)$ could be determined for *Am*PDH. A slope close to 0.5 indicates a one‐electron reduction step, which was observed for variant H103A and H556A. Hence, both one‐electron reduction steps could be determined for these variants. *M*, maximum fraction of thermodynamically stable semiquinone formed, which was necessary to calculate $E^{o\prime }$ for both variants

Variant	$E^{o\prime }\left(\text{mV}\right)$	$E_{1}^{o\prime }\left(\text{mV}\right)$	$E_{2}^{o\prime }\left(\text{mV}\right)$	Slope	*M*
*Am*PDH	+92	–	–	0.97	–
H103A	+63	+113	+13	0.41	0.78
H556A	+84	+129	+39	0.50	0.74

For H556A (Fig. 8C and Table 7), similar observations as for H103A were made. The fully oxidized enzyme was reduced by two one‐electron reduction steps, as indicated by a slope of 0.50 when plotting log(*E*_red_/*E*_ox_) *versus* log(*D*_red_/*D*_ox_). This again points towards a putative semiquinone intermediate on the way to the fully reduced enzyme. The observed intermediate for variant H556A had an absorption maximum at 368 nm (Fig. 8C, dashed line) and was detected previously in the resting state of *Am*PDH, which could be attributed to an equilibrium of the oxidized form of the enzyme with its reduced forms 20. For variant H556A, $E^{o\prime }$ was +84 ± 4 mV at pH 7.0 and 25 °C (Table 7). Therefore, the redox potential of variant H556A is only decreased by 8 mV compared to *Am*PDH, indicating that the difference in redox potential between H556A and *Am*PDH is not responsible for the 720‐fold decrease of the reductive half‐reaction with GLC (see above).

**Figure 9:**
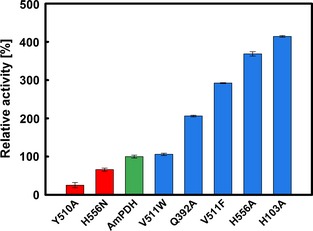
Relative oxygen reactivities of wild‐type recombinant pyranose dehydrogenase from *A. meleagris* and active‐site variants. Steady‐state oxygen reactivity was determined using an Amplex Red/horseradish peroxidase based assay 19. The assay contained 0.5 mg·mL ^−1^ of the purified enzyme, 50 μm Amplex Red, 25 mm GLC, 0.1 U·mL ^−1^ horseradish peroxidase and 50 mm sodium phosphate buffer (pH 7.4). Error bars represent the SD of four repeats. The specific activity of wild‐type recombinant *Am* PDH with oxygen is 0.104 μm·min^−1^·mg^−1^ (green bar).

It is not exactly known why flavoenzymes do or do not react with oxygen 19, 44. Factors such as a positive charge in the active site 45, its accessibility via channels and tunnels 46 and the microenvironment around the flavin N5‐C(4a) 47, 48 have been identified as important features for oxygen reactivity. Therefore, all generated variants were routinely probed for their steady‐state oxygen reactivity, with variant H512A being the only exception because it reacted too poorly with the electron donor GLC. Results for relative oxygen activities are given in Fig. 9. Wild‐type *Am*PDH had a very low oxygen reactivity of 0.104 μm·min^−1^·mg^−1^. Several of the variants showed an increase in oxygen reactivity, yet, despite this increase, the reaction with oxygen is still poor. The highest oxygen reactivity amongst all tested variants (0.430 μm·min^−1^·mg^−1^, corresponding to 414% of wild‐type *Am*PDH) was found for H103A, which lacks the covalent bond to FAD and showed a significantly reduced redox potential. This is in line with previously published work on variant H103Y, which was found to have a 5.3‐fold higher oxygen reactivity compared to *Am*PDH 19. The oxygen reactivity for variant H556A was increased to a similar level as for H103A; however, its redox potential was only slightly decreased compared to *Am*PDH. Consequently, a direct link between lower redox potential and higher oxygen reactivity cannot be drawn in *Am*PDH. Moreover, one would expect rather the opposite correlation, namely a high redox potential that is linked to increased oxygen reactivity, because a higher redox potential is usually associated with electron accepting redox‐partners that have high redox potentials, such as oxygen.

## Conclusions

We investigated *Am*PDH and eight variants thereof by biochemical, biophysical and computational means. The secondary structures and thermostability were not affected by the newly introduced amino acids as judged by ECD spectroscopy and *Thermo*FAD experiments, respectively. All enzymes were monocovalently flavinylated, and only variant H103A had a noncovalently but tightly bound FAD cofactor. The redox potential for this variant was decreased by 29 mV, which is in line with other flavin‐dependent oxidoreductases 30. Apparent steady‐state kinetics demonstrated that His512 is the sole catalytic base in the reductive half‐reaction of *Am*PDH, whereas the second active‐site histidine, His556, is mainly involved in substrate interaction. Consequently, the substrate specificity of *Am*PDH cannot be explained by the presence of two active‐site histidines acting as a general base and must be attributed to other factors. Predictions from MD simulations, which were verified by GC‐MS measurements, unequivocally demonstrated that *Am*PDH and its variants preferentially oxidize GLC *via* 2‐keto‐GLC to the final product 2,3‐diketo‐GLC.

## Materials and methods

### Chemicals and vectors

All chemicals were of the highest available purity and purchased from Sigma‐Aldrich (St Louis, MO, USA), VWR (Radnor, PA, USA) and Roth (Karlsruhe, Germany). Primers were obtained from LGC Genomics (Vienna, Austria) or Sigma‐Aldrich. Restriction endonucleases, T4 DNA ligase and Phusion polymerase were obtained from Thermo Fisher Scientific Biosciences (St Leon‐Rot, Germany). GoTaq polymerase was purchased from Promega (Madison, WI, USA). Zeocin and the pPICZB vector were obtained from Invitrogen (Carlsbad, CA, USA). The 2‐keto‐GLC standard, meso‐erythritol, LC‐MS grade distilled water, ethoxylamine hydrochloride, water‐free pyridine and *N*‐methyl‐*N*‐(trimethylsilyl)trifluoroacetamide with 1% trimethylchlorosilane in water‐free pyridine were purchased from Sigma‐Aldrich.

### Strains and media

Strains and media used for the present study were essentially the same as reported previously 19. In short, *Escherichia coli* strain NEB5α was obtained from New England Biolabs (Ipswich, MA, USA) and *P. pastoris* strain X33 was obtained from Invitrogen. YPD plates contained 20 g·L^−1^ peptone from casein, 10 g·L^−1^ yeast extract, 4 g·L^−1^ GLC, 15 g·L^−1^ agar and 100 mg·L^−1^ Zeocin. LB low‐salt (LB‐LS) plates contained 10 g·L^−1^ peptone from casein, 5 g·L^−1^ yeast extract, 10 g·L^−1^ NaCl, 15 g·L^−1^ agar and 25 mg·L^−1^ Zeocin. For fermenter cultivations, the basal salts medium with 4.35 mL·L^−1^ PTM_1_ trace salts was used, in accordance with the manufacturer's instructions (Invitrogen) and as described in detail previously 19.

### Plasmid construction for expression in *P. pastoris*

When expressing a protein of interest with the original pPICZB plasmid from Invitrogen, a *myc*‐epitope followed by a His_6_‐tag is added to the C‐terminus of the protein. To alter the protein of interest as little as possible, the pPICZB plasmid was slightly modified for the present study. When using the primer pair pPICZB‐6His‐fw and pPICZB‐6His‐*Xba*I‐rv (Table S1) the *myc*‐epitope was removed using the *Dpn*I‐method 49, creating pPICZB‐His_6_. The PCR‐product was purified from an agarose gel, and digested with *Dpn*I for 2 h at 37 °C for degradation of methylated template DNA. After another purification step, the pPICZB vector void of the *myc*‐epitope was transformed into chemically competent *E. coli* NEB5α. Positive transformants were selected on LB‐LS plates containing Zeocin and subsequently sequenced. The confirmed plasmid was amplified in *E. coli* NEB5α, purified, and stored at –20 °C.

The *A. meleagris pdh1* (*ampdh*) gene in the pPICZB vector as used by Krondorfer *et al*. 19 was amplified with the primer pair *Am*PDH‐*Not*I‐fw and *Am*PDH‐*Xba*I‐rv (Table S1), thereby introducing a 3′‐*Not*I‐ and a 5′‐*Xba*I‐restriction site. The purified product was digested with *Not*I and *Xba*I, and ligated into the equally treated pPICZB‐His_6_ vector, yielding pPICZB‐His_6_‐*Am*PDH, which was transformed into chemically competent *E. coli* NEB5α. Positive transformants were selected on LB‐LS plates containing Zeocin and subsequently sequenced. An *E. coli* NEB5α colony containing the verified plasmid was proliferated, pPICZB‐His_6_‐*Am*PDH purified and finally stored at –20 °C.

The active site mutations H103A, Q392A, Y510A, V511F, V511W, H512A, H556A and H556N were each introduced into a separate pPICZB‐His_6_‐*Am*PDH vector by site‐directed mutagenesis. The overlapping primer pairs for introducing the respective mutations were H103A‐fw/H103A‐rv, Q392A‐fw/Q392A‐rv, Y510A‐fw/Y510A‐rv, V511F‐fw/V511‐rv, V511W‐fw/V511‐rv, H512A‐fw/H512A‐rv, H556Afw/H556A‐rv and H556N‐fw/H556N‐rv (Table S1). The mutations were verified by sequencing. Plasmids with the correct mutation were linearized with *Pme*I at 37 °C for 2 h, purified and transformed into electro‐competent *P. pastoris* X33. Positive transformants were selected on YPD plates containing Zeocin. Additionally, plasmid integration was tested by colony PCR, for which the universal primers 5′‐AOX1 and 3′‐AOX1 were employed.

### Protein expression and purification

Recombinant wild‐type *A. meleagris* PDH1 (*Am*PDH) and the H556A variant were produced as described previously 3 but in a 42 L computer‐controlled stirred tank reactor (Applikon, Schiedam, The Netherlands) with an initial volume of 20 L of basal salts fermentation medium. The variants H103A, Q392A, Y510A, V511F, V511W, H512A and H556N were produced as described previously 19 in a 7 L computer‐controlled fermenter (MBR, Wetzikon, Switzerland) with an initial volume of 4 L of basal salts fermentation medium.

*Am*PDH and H556A were purified at room temperature according to a four‐step purification scheme. First, the volume of the cultivation supernatant was reduced by a factor of approximately 10 employing cross‐flow filtration (Microza ultrafiltration module; Pall, Vienna, Austria) using a membrane with a 10 kDa cut‐off. The concentrate was subsequently purified by IMAC with a Ni^2+^‐charged Chelating Sepharose Fast Flow column (65 mL; flow rate 10 mL·min^−1^; GE Healthcare, Little Chalfont, UK), equilibrated with 100 mm potassium phosphate buffer (pH 7.0), 1 m NaCl and 5 mm imidazole. After a washing step of 3 column volumes (CV), *Am*PDH or H556A were eluted with a linear gradient from 5 to 500 mm imidazole. Fractions with PDH activity were pooled. (NH_4_)_2_SO_4_ was added to the pooled IMAC I‐fractions to 40% saturation and loaded onto a Phenyl Sepharose Fast Flow column (200 mL; flow rate 10 mL·min^−1^; GE Healthcare) equilibrated with 50 mm potassium phosphate buffer [pH 6.5, 40% saturation (NH_4_)_2_SO_4_]. After washing the column with 3 CV of the same buffer, proteins were eluted with a linear gradient of starting buffer to 50 mm potassium phosphate buffer (pH 6.5) in 1 CV. Fractions with PDH activity were pooled. As a polishing step, a second IMAC purification was conducted. The pooled hydrophobic interaction chromatography fractions were loaded onto four in‐series connected HisTrap HP columns (5 mL each; flow rate 2 mL·min^−1^; GE Healthcare) equilibrated as for IMAC I. For washing and elution, conditions the same as those for IMAC I were applied. Fractions with PDH activity were pooled. The variants H103A, Q392A, Y510A, V511F, V511W, H512A and H556N were purified using a combination of hydrophobic interaction chromatography and IMAC II as described above.

Imidazole from the last IMAC step was removed for all purified enzymes by ultrafiltration (10 kDa cut‐off membrane; Amicon Ultra Centrifugal Filter Device; Millipore, Billerica, MA, USA). The purified and concentrated enzymes were washed thrice with 10 mL of 50 mm potassium phosphate buffer (pH 7.0). *Am*PDH is mainly isolated in its reduced form 13, 19. To fully oxidize *Am*PDH and its variants, the enzymes were re‐oxidized with 5 mm 2,6‐dichlorophenol‐indophenol (DCIP). The solution was subsequently passed through a PD‐10 desalting column (GE Healthcare) equilibrated with 50 mm potassium phosphate buffer pH 7.0 to remove DCIP. Then, the solution was sterile‐filtered by passing through a membrane of 0.22 μm cut‐off (Merck Millipore, Darmstadt, Germany), and finally diluted in sterile potassium phosphate buffer pH 7.0 to a protein concentration of approximately 20–50 mg·mL^−1^. Aliquots of 100 μL were shock‐frozen in liquid nitrogen and stored at –80 °C. All variants were stored at 4 °C after sterile filtration.

### Enzyme activity assay and molecular properties

PDH activity was determined by following the reduction of the ferrocenium ion (Fc^+^) at 300 nm and 30 °C for 3 min as described previously 50 with certain adaptions: the standard 1 mL of reaction mixture contained 50 μmol sodium phosphate buffer (pH 7.5), 25 μmol GLC and 0.2 μmol of ferrocenium hexafluorophosphate. Protein concentrations were determined according to Bradford with a pre‐fabricated assay (Bio‐Rad, Hercules, CA, USA). SDS/PAGE and enzymatic deglycosylation with PNGase F were conducted as described previously 2, 19.

Determination of molar absorption coefficients for *Am*PDH, H103A, and H556A were performed using a protocol adapted from a previous study 27. In brief, the enzyme solution was diluted in 50 mm potassium phosphate buffer (pH 7.0) to an absorbance at 450 nm of approximately 2.5. To 100 μL of that solution, either 900 μL of the same buffer (for spectra of intact enzyme) or 900 μL of 6 m guanidine HCl (for spectra of denatured enzyme) was added. Concentrations of free FAD released from the denatured enzymes were determined in triplicate based on the free FAD molar absorption coefficient (ε_450_ = 1.1 × 10^4^ m ^−1^·cm^−1^). The extinction coefficients of FAD bound to the polypeptides were ε_463_ = 9.5 × 10^3^ m ^−1^·cm^−1^ for *Am*PDH, ε_460_ = 1.2 × 10^4^ m ^−1^·cm^−1^ for H103A and ε_465_ = 1.1 × 10^4^ m ^−1^·cm^−1^ for H556A. The low ε_463_ for *Am*PDH suggests a deprotonated flavin N3‐atom 29, 51.

### ECD spectroscopy

To compare the overall fold and secondary structure elements of *Am*PDH and its variants, far UV ECD spectra were recorded as described previously 20 from 180 to 260 nm on a Chirascan CD Spectrophotometer (Applied Photophysics, Leatherhead, UK), which was flushed with nitrogen. The cuvette pathlength was 1 mm, the spectral bandwidth was set to 3 nm and the scan time per point to 10 s. The protein concentration was adjusted to approximately 4 μm with 50 mm potassium phosphate buffer (pH 7.0).

### *Thermo*FAD

*Thermo*FAD measurements were conducted to elucidate the thermal unfolding temperature *T*_m_ 25, utilizing the increased intrinsic fluorescence of the FAD cofactor upon thermal protein denaturation. Two replica were measured, each containing 25 μL with 65 μm *Am*PDH or the respective variant in 40 mm Britton‐Robinson buffer (pH 2–9, in steps of 1 pH units). Heating of samples was conducted from 20–95 °C in steps of 0.5 °C·s^−1^ with an iCycler Thermal Cycler equipped with an MyiQ Real‐Time PCR Optical Module (Bio‐Rad), and the corresponding fluorescence was recorded. *T*_m_ was determined from the maximum of the first derivative of the obtained sigmoidal curve 25, 52.

### TCA/acetone precipitation

To determine whether FAD is covalently attached to the polypeptide, a solution of oxidized enzyme with an absorbance (450 nm) between 0.1–0.3 was prepared in 50 mm potassium phosphate buffer (pH 7.0) and the oxidized spectrum was subsequently recorded from 250 to 650 nm (U‐3000 spectrophotometer; Hitachi, Tokyo, Japan). Precipitation was conducted as described previously 19 by mixing the double‐concentrated protein solution with 10% (v/v) TCA and 40% (v/v) acetone and incubation for 10 min on ice. After centrifugation at 17 400 ***g*** for 5 min at 4 °C, the spectra of the supernatants were recorded to identify the presence of noncovalently bound FAD.

### Steady‐state kinetics

Apparent kinetic constants for the electron donor GLC were measured utilizing the standard Fc^+^ assay. Data for Fc^+^ were collected in 100 mm borate buffer (pH 8.5) and the concentration of the electron donor GLC was adjusted according to the *K*_M_ value of the corresponding variant: 25 mm (ns*Am*PDH, rec*Am*PDH and *Am*PDH), 50 mm (H103A, Q392A and V511W), 250 mm (Y510A) and 500 mm (H556A, H556N and V511F). The data obtained were fitted to the Michaelis–Menten equation by nonlinear least squares regression with sigma plot, version 11 (Systat Software, Chicago, IL, USA), from which the kinetic constants were derived. Catalytic efficiencies were used to calculate difference in transition state energies ∆∆*G*_ES‡_ between variants and wild‐type *Am*PDH 53, 54 according to:

(1) $$\Delta \Delta G_{\mathrm{ES}}{}^{‡}=\text{RT}\text{ln}[\frac{{\left(k_{\mathrm{cat}}/K_{M}\right)}_{\mathrm{var}}}{{\left(k_{\mathrm{cat}}/K_{M}\right)}_{\mathrm{wt}}}]$$

### MD simulations and free energy calculations

The structure preparations were essentially performed as described previously 17. In brief, two different binding poses (pose A and B) of GLC were considered, differing in a rotation of 180° about the axis defined by a line running through a point midway between the GLC atoms C5 and O5, and a point midway between atoms C2 and C3, allowing for GLC oxidation at C2 or C3. Only two protonation states of the active‐site histidines were considered, which had the most favourable binding energies and highest ligand stabilities of GLC in *Am*PDH in a previously reported comparison of the protonation states 17. For pose A (Fig. 1), His512 and His556 were both fully protonated (protonation state PP); for pose B, His512 was fully protonated and His556 was in its neutral state with a proton at Nε (protonation state PN). For *in silico* mutations to more bulky side chains (V511F and V511W), rotamers most similar to the wild‐type were selected. During the 10 ns simulations, the side chains of these variants could rotate and adopt new conformations. For *in silico* mutations to less bulky residues, initial atom positions of the side chains were derived from the wild‐type residues.

The simulation set‐up was carried out as described previously 17. The MD simulations were conducted with gromos, version 11 55 employing the 53A6 force field 56 in explicit solvent within a rectangular, periodic and pre‐equilibrated box of SPC water 57. After energy minimization and equilibration 58, two independent 10 ns production runs (md1 and md2) were performed at constant pressure (1 atm) and temperature (300 K) using the weak‐coupling scheme 59 with coupling times of 0.5 and 0.1 ps, respectively. The isothermal compressibility was set to 4.575 × 10^–4^ kJ^−1^·mol·nm^−3^, and two separate temperature baths were used for solute and solvent. The SHAKE algorithm was applied to constrain bond lengths 60 for solute and solvent allowing for 2 fs time‐steps. Nonbonded interactions were calculated using a triple range scheme. Interactions within a short‐range cut‐off of 0.8 nm were calculated at every time step from a pair list that was updated every fifth step. At these points, interactions between 0.8 nm and 1.4 nm were also calculated explicitly and kept constant between updates. A reaction field 61 contribution was added to the electrostatic interactions and forces to account for a homogenous medium outside the long‐range cut‐off using a relative dielectric constant of 61 as appropriate for the SPC water model 62. Coordinate and energy trajectories were stored every 0.5 ps for subsequent analysis.

Binding free energies (Δ*G*_bind_) relative to *Am*PDH (ΔΔ*G*_bind_) were estimated from experimental *K*_M_ values $\left(\Delta \Delta G_{\mathrm{bind}}^{\exp}\right)$ according to:

(2) $$\Delta \Delta G_{\mathrm{bind}}^{\exp}=k_{B}T\ln\left[\frac{K_{M}^{\mathrm{variant}}}{K_{M}^{Am\mathrm{PDH}}}\right]$$

where *k*_B_ *T* is the Boltzmann constant multiplied with the absolute temperature, or calculated from MD simulations $\left(\Delta \Delta G_{\mathrm{bind}}^{\mathrm{sim}}\right)$ by employing the linear interaction energy method 63 according to the equation 64:

(3) $$\Delta G_{\mathrm{bind}}^{\mathrm{sim}}=\beta \left({\left\langle V_{\mathrm{lig}-\mathrm{sur}}^{\mathrm{EL}}\right\rangle }_{\mathrm{protein}}-{\left\langle V_{\mathrm{lig}-\mathrm{sur}}^{\mathrm{EL}}\right\rangle }_{\mathrm{free}}\right)+\alpha \left({\left\langle V_{\mathrm{lig}-\mathrm{sur}}^{\mathrm{VdW}}\right\rangle }_{\mathrm{protein}}-{\left\langle V_{\mathrm{lig}-\mathrm{sur}}^{\mathrm{VdW}}\right\rangle }_{\mathrm{free}}\right)$$

where angular brackets indicate ensemble averages, calculated over a simulation of the substrate bound to the protein (protein) or free in solution (free). $V_{\mathrm{lig}-\mathrm{sur}}^{\mathrm{EL}}$ and $V_{\mathrm{lig}-\mathrm{sur}}^{\mathrm{VdW}}$ represent the ligand‐surrounding electrostatic and van der Waals interactions, respectively. α and β are parameters of the linear interaction energy equation. $\Delta \Delta G_{\mathrm{bind}}^{\mathrm{sim}}$ was obtained by subtracting $\Delta G_{\mathrm{bind}}^{\mathrm{sim}}$ of wild‐type *Am*PDH from $\Delta G_{\mathrm{bind}}^{\mathrm{sim}}$ of the respective variant. The different poses (pose A and B) and runs (md1 and md2) were averaged as described previously 65.

### Analysis of GLC reaction products by GC‐MS

Stock solutions of glucose, 2‐keto‐glc and meso‐erythritol (internal standard) were prepared by dissolving appropriate amounts of solid standard in LC‐MS grade water in amber LC vials and were kept at −40 °C. Working solutions were prepared daily by diluting the stock solution in LC‐MS grade water. The ethoxymation solution was prepared daily by dissolving 18.7 mg of ethoxylamine hydrochloride in 1 mL of water‐free pyridine. A nonpolar HP‐1MS column (60 m length × 250 µm inner diameter × 0.25 µm thickness film; 100% dimethylpolysiloxane) and a guard column (5 m length × 320 µm innner diametter without stationary phase) from Agilent Technologies were used for separation. EZ‐2 Envi SpeedVAC from Genevac Inc (Stone Ridge, NY, USA) was used for drying the standards and samples. GC‐MS was carried out with an Agilent Technologies GC‐EI‐MS machine consisting of an Agilent Technologies 7820A gas chromatograph and an Agilent Technologies 5975 mass selective detector. GC‐CI‐ToFMS was conducted on an Agilent Technologies 7200 GC‐CI/EI‐QToFMS. Sample preparation and derivatization was performed as described previously 66.

The derivatization product solution was kept at 4 °C in an auto‐sampler, of which 1 µL was injected into a nonpolar HP‐1MS column with an Agilent Technologies guard column with pulsed injection mode at 30 psi. The temperature of the injector and the transfer line were kept constant at 250 °C and 280 °C, respectively. Helium (99.9999% purity grade) was used as carrier gas with a flow rate of 1.2 mL·min^−1^ in constant flow mode. The column oven was kept at 70 °C for 1 min and heated up to 200 °C (20 °C·min^−1^), then increased to 240 °C (2.5 °C·min^−1^) and then finally to 310 °C (20 °C·min^−1^), where it was kept for 1 min to re‐condition the column. For GC‐CI‐QToFMS, temperature, pressure of methane gas, emission current, electron energy and QToFMS parameters were optimized as reported previously 67. The mass range in both cases was set to 70–700 amu. An *n*‐alkane (C8‐C40) retention index in ethoxylamine and *N*‐methyl‐*N*‐(trimethylsilyl)trifluoroacetamide solution was injected into GC‐EI‐MS to calculate the modified retention indices according to 68. Cross‐check mass fragmentation, determiniation of the electron ionization mass spectra pathway and chemical ionization were carried out using mass frontier, version 7.0 SP1 (Thermo Scientific). Quantification of 2‐keto‐glc was carried out with external standards. For 3‐keto‐glc and 2,3‐diketo‐glc no standards were commercially available. Therefore, semiquantitative analysis was performed by comparing the peak areas of the corresponding derivatization products with those of 2‐keto‐glc at the same mass‐to‐charge ratio.

### Rapid reaction experiments

Reactions were carried out as described previously 69. In brief, 50 mm potassium phosphate buffer (pH 7.0) at 4 °C, a TgK Scientific model SF‐61DX or a TgK Scientific model SHU‐61SX2 (TgK Scientific, Bradford‐on‐Avon, UK) stopped‐flow spectrophotometer in single‐mixing mode and a dead‐time of 2 ms were used. The stopped‐flow instrument was made anaerobic by flushing the flow system with an anaerobic buffer solution containing 0.5 mg·mL^−1^ dithionite in 50 mm potassium phosphate buffer (pH 7.0) and equilibrated in that solution overnight. Before starting the experiments, the flow system of the instrument was washed three times with anaerobic 50 mm potassium phosphate buffer (pH 7.0).

The enzymes obtained from purification contained partially reduced populations. To prepare fully oxidized enzymes, 5 mm DCIP was used as described above. To prepare a reduced enzyme solution, an anaerobic oxidized enzyme solution was stoichiometrically reduced in an anaerobic glove box with a 300 μm GLC solution in potassium phosphate buffer (pH 7.0), which was monitored spectrophotometrically. All substrate concentrations used were more than five‐fold excess of the enzyme concentration (30 μm) to ensure pseudo first‐order conditions.

To study the kinetic reduction of wild‐type *Am*PDH by sugar substrates, the reaction was performed aerobically because the enzyme was shown before to be reoxidized very slowly with oxygen 13. Anaerobic conditions were used for stopped‐flow experiments of variant H556A, which was shown to have a 3.7‐fold elevated oxygen reactivity compared to *Am*PDH. Reoxidation experiments with 1,4‐benzoquinone were also performed anaerobically to prevent side‐reactions of 1,4‐benzoquinone with oxygen.

Observed rate constants (*k*_obs_) for the fast and slow phase were calculated from double‐exponential fits to kinetic traces using the kinetic studio (TgK Scientific) and program a (R. Chang, J.‐Y. Chiu, J. Dinverno and D. P. Ballou, University of Michigan, MI, USA). The *k*_obs_ values obtained were plotted against the substrate concentrations and the apparent bimolecular rate constant (*k*_app_) was calculated from the slope. Simulations were performed by numerical methods with Runge‐Kutta algorithms implemented in berkeley madonna, version 8.3 (University of Berkley, CA, USA) with a time step of 2 × 10^–4^ s. The kinetic models used to simulate the reductive and oxidative half‐reaction are listed in the Results.

### Redox potential determination

The redox potentials of *Am*PDH, H103A, and H556A were measured at 25 °C according to the method of Massey 70 using xanthine and xanthine oxidase as the reduction system 7, 71. The enzymes were fully oxidized with 5 mm DCIP as described above. Solutions of fully oxidized enzyme (*Am*PDH, H103A or H556A), the standard dye benzyl viologen, xanthine and xanthine oxidase (side arm) were pipetted together to a final volume of 1 mL in a specially designed cuvette equipped with two side arms and a stopcock. Anaerobiosis was established by repeated cycles of evacuation and flushing with oxygen‐free nitrogen. Subsequently, the reaction was started by adding xanthine oxidase from the side arm. The reduction was monitored by recording spectra with an Agilent Technologies 8453 diode array spectrophotometer.

Phenazine methosulfate ($E^{o\prime }=80\text{mV}$) was used as standard dye for *Am*PDH. Enzyme and dye were monitored at 463 nm (*Am*PDH) and 387 nm (phenazine methosulfate) during their slow reduction over a period of approximately 8 h. *Am*PDH has no isosbestic point at 387 nm but a significant absorbance, which changes with altering redox states of the enzyme. Phenazine methosulfate has a small absorbance at 463 nm, which hardly changes during the reduction process of the dye. Consequently, the absorbance at 463 nm and 387 nm had to be analysed with respect to a mixed population of enzyme and dye. For this, the equations used were:

(4) $$\varepsilon_{463\mathrm{nm}}^{E}\;c^{E}\;+\;\varepsilon_{463\mathrm{nm}}^{D}\;c^{D}=A_{463\mathrm{nm}}$$

(5) $$\varepsilon_{387\mathrm{nm}}^{E}\;c^{E}\;+\;\varepsilon_{387\mathrm{nm}}^{D}\;c^{D}=A_{387\mathrm{nm}}$$

where superscript E represents ‘Enzyme’ and D represents ‘Dye’ at the wavelength indicated in the subscript, ε represents the extinction coefficient, and *c* is the (unknown) concentration. This system of two equations was solved to yield the concentrations of the enzyme or dye. These concentrations were subsequently used to calculate the ratio of reduced and oxidized species at various stages during the reduction process. The midpoint potential $\left(E^{o\prime }\right)$ of *Am*PDH was determined using the standard Nernst equation as described previously 72.

For variants H103A and H556A, methylene blue $\left(E^{o\prime }=11\text{mV}\right)$ was used as standard dye. The variants and dye were monitored at 460 nm (H103A), 465 nm (H556A) and 664 nm (methylene blue), where the monitored absorbance of variant H103A or H556A did not interfere with the absorbance of the dye and vice versa. Consequently, the concentrations of dye and variant could be readily obtained by the absorbance at the indicated wavelength. For both variants, these concentrations were used to calculate the ratio of reduced and oxidized species at various stages during the reduction process. Because an intermediate species with an absorbance maximum of 422 nm (H103A) or 368 nm (H556A) was observed for both variants, subsequent calculations employing the standard Nernst equation 72 yielded the semiquinone/reduced enzyme half potential ($E_{2}^{o\prime }$). $E_{2}^{o\prime }$ was used to calculate $E^{o\prime }$ for both variants according to the equations 71, 73, 74:

(6) $$E_{1}^{o\prime }-E_{2}^{o\prime }=2\left(\frac{2.303\mathrm{RT}}{F}\right)\log\left[\frac{2M}{\left(1-M\right)}\right]$$

$$E_{1}^{o\prime }+E_{2}^{o\prime }=2E^{o\prime }$$

where *M* is the maximum fraction of thermodynamically stable semiquinone formed, *T* is 298 K, $E^{o\prime }$ is the two‐electron reduction potential, $E_{1}^{o\prime }$ is the oxidized enzyme/semiquinone half potential and $E_{2}^{o\prime }$ is the semiquinone/reduced enzyme half potential. The amount of thermodynamically stable semiquinone (*M*) was quantified in mixtures with compositions similar to those described above. In these mixtures, the standard dye was replaced by 50 μL of a 2 mm allopurinol solution, a potent xanthine oxidase inhibitor 75, which was pipetted into the second side arm of the cuvette. The xanthine and xanthine oxidase reduction system was stopped after semiquinone absorption reached its maximum (*SQ*_MAX_) at 422 nm (H103A) or 368 nm (H556A) by adding the allopurinol solution from the side arm of the cuvette. The absorbance at 422 nm (H103A) or 368 nm (H556A) was recorded again after letting the reaction mixture stand at room temperature in the dark overnight (*SQ*_ON_). Finally, *M* was obtained by calculating *SQ*_ON_/*SQ*_MAX_.

### O_2_ reactivity

The steady‐state oxygen reactivity for all purified enzymes was determined in quadruplicate with a fluorimetric Amplex Red/horseradish peroxidase assay using the calibration curve described previously 19. In the present study, 25 and 100 μm of the electron donor GLC were used to compensate for rather high apparent *K*_M,GLC_ values for some variants. The obtained oxygen reactivities were essentially the same for both GLC concentrations. Therefore, only oxygen reactivities for 25 μm GLC are reported. The measured H_2_O_2_ generation (min^−1^·mg^−1^ enzyme) was converted via the calibration curve into the oxygen reactivity, which is given as μm O_2_ min^−1^·mg^−1^ enzyme.

## Author contributions

COo, DH, GK, MMHG, PC, SH and UB planned the experiments. DBC, JS and MMHG performed the experiments. CKP, CO, COo, DH, GK, JS, MMHG, PC, PGF, SH and UB performed the data analysis and interpretation. CO, COo, DH, MMHG and PC wrote the paper.

## Supporting information


**Table S1.** Nucleotide sequences of the primers used.

## References

[1] Peterbauer CK & Volc J (2010) Pyranose dehydrogenases: biochemical features and perspectives of technological applications. Appl Microbiol Biotechnol 85, 837–848. 19768457 10.1007/s00253-009-2226-y

[2] Sygmund C , Kittl R , Volc J , Halada P , Kubátová E , Haltrich D & Peterbauer CK (2008) Characterization of pyranose dehydrogenase from *Agaricus meleagris* and its application in the C‐2 specific conversion of d‐galactose. J Biotechnol 133, 334–342. 18083263 10.1016/j.jbiotec.2007.10.013

[3] Sygmund C , Gutmann A , Krondorfer I , Kujawa M , Glieder A , Pscheidt B , Haltrich D , Peterbauer C & Kittl R (2012) Simple and efficient expression of *Agaricus meleagris* pyranose dehydrogenase in *Pichia pastoris* . Appl Microbiol Biotechnol 94, 695–704. 22080342 10.1007/s00253-011-3667-7 PMC3315643

[4] Hernández‐Ortega A , Lucas F , Ferreira P , Medina M , Guallar V & Martínez AT (2012) Role of active site histidines in the two half‐reactions of the aryl‐alcohol oxidase catalytic cycle. Biochemistry 51, 6595–6608. 22834786 10.1021/bi300505z

[5] Fan F & Gadda G (2005) On the catalytic mechanism of choline oxidase. J Am Chem Soc 127, 2067–2074. 15713082 10.1021/ja044541q

[6] Müller D (1928) Oxidation von Glukose mit Extrakten aus *Aspergillus niger* . Biochem Z 199, 136–170.

[7] Kujawa M , Ebner H , Leitner C , Hallberg BM , Prongjit M , Sucharitakul J , Ludwig R , Rudsander U , Peterbauer C , Chaiyen P *et al* (2006) Structural basis for substrate binding and regioselective oxidation of monosaccharides at C3 by pyranose 2‐oxidase. J Biol Chem 281, 35104–35115. 16984920 10.1074/jbc.M604718200

[8] Cavener DR (1992) GMC oxidoreductases: a newly defined family of homologous proteins with diverse catalytic activities. J Mol Biol 223, 811–814. 1542121 10.1016/0022-2836(92)90992-s

[9] Wongnate T & Chaiyen P (2013) The substrate oxidation mechanism of pyranose 2‐oxidase and other related enzymes in the glucose–methanol–choline superfamily. FEBS J 280, 3009–3027. 23578136 10.1111/febs.12280

[10] Volc J , Kubátová E , Wood DA & Daniel G (1997) Pyranose 2‐dehydrogenase, a novel sugar oxidoreductase from the basidiomycete fungus *Agaricus bisporus* . Arch Microbiol 167, 119–125. 9133318

[11] Volc J , Kubátová E , Daniel G , Sedmera P & Haltrich D (2001) Screening of basidiomycete fungi for the quinone‐dependent sugar C‐2/C‐3 oxidoreductase, pyranose dehydrogenase, and properties of the enzyme from *Macrolepiota rhacodes* . Arch Microbiol 176, 178–186. 11511865 10.1007/s002030100308

[12] Volc J , Sedmera P , Halada P , Daniel G , Přikrylová V & Haltrich D (2002) C‐3 oxidation of non‐reducing sugars by a fungal pyranose dehydrogenase: spectral characterization. J Mol Catal – B Enzym 17, 91–100.

[13] Tan TC , Spadiut O , Wongnate T , Sucharitakul J , Krondorfer I , Sygmund C , Haltrich D , Chaiyen P , Peterbauer CK & Divne C (2013) The 1.6 Å crystal structure of pyranose dehydrogenase from *Agaricus meleagris* rationalizes substrate specificity and reveals a flavin intermediate. PLoS ONE 8, e53567. 23326459 10.1371/journal.pone.0053567 PMC3541233

[14] Sedmera P , Halada P , Peterbauer C & Volc J (2004) A new enzyme catalysis: 3,4‐dioxidation of some aryl β‐d‐glycopyranosides by fungal pyranose dehydrogenase. Tetrahedron Lett 45, 8677–8680.

[15] Volc J , Sedmera P , Kujawa M , Halada P , Kubátová E & Haltrich D (2004) Conversion of lactose to β‐d‐galactopy‐ranosyl‐(1→4)‐d‐arabino‐hexos‐2‐ulose‐(2‐dehydro‐lactose) and lactobiono‐1,5‐lactone by fungal pyranose dehydrogenase. J Mol Catal B Enzym 30, 177–184.

[16] Sedmera P , Halada P , Kubátová E , Haltrich D , Přikrylová V & Volc J (2006) New biotransformations of some reducing sugars to the corresponding (di)dehydro(glycosyl) aldoses or aldonic acids using fungal pyranose dehydrogenase. J Mol Catal B Enzym 41, 32–42.

[17] Graf MMH , Bren U , Haltrich D & Oostenbrink C (2013) Molecular dynamics simulations give insight into d‐glucose dioxidation at C2 and C3 by *Agaricus meleagris* pyranose dehydrogenase. J Comput Aided Mol Des 27, 295–304. 23591812 10.1007/s10822-013-9645-7 PMC3657087

[18] Graf MMH , Lin Z , Bren U , Haltrich D , van Gunsteren WF & Oostenbrink C (2014) Pyranose dehydrogenase ligand promiscuity: a generalized approach to simulate monosaccharide solvation, binding, and product formation. PLoS Comput Biol 10, e1003995. 25500811 10.1371/journal.pcbi.1003995 PMC4263366

[19] Krondorfer I , Lipp K , Brugger D , Staudigl P , Sygmund C , Haltrich D & Peterbauer CK (2014) Engineering of pyranose dehydrogenase for increased oxygen reactivity. PLoS ONE 9, e91145. 24614932 10.1371/journal.pone.0091145 PMC3948749

[20] Krondorfer I , Brugger D , Paukner R , Scheiblbrandner S , Pirker KF , Hofbauer S , Furtmüller PG , Obinger C , Haltrich D & Peterbauer CK (2014) *Agaricus meleagris* pyranose dehydrogenase: influence of covalent FAD linkage on catalysis and stability. Arch Biochem Biophys 558, 111–119. 25043975 10.1016/j.abb.2014.07.008 PMC4148704

[21] Tan TC , Haltrich D & Divne C (2011) Regioselective control of β‐d‐glucose oxidation by pyranose 2‐oxidase is intimately coupled to conformational degeneracy. J Mol Biol 409, 588–600. 21515286 10.1016/j.jmb.2011.04.019

[22] Cremata JA , Montesino R , Quintero O & García R (1998) Glycosylation profiling of heterologous proteins In Pichia Protocols (Higgins DR & Gregg JM, eds), pp. 95–105. Humana Press, Totowa, NJ. 10.1385/0-89603-421-6:95 9680636

[23] Kelly SM , Jess TJ & Price NC (2005) How to study proteins by circular dichroism. Biochim Biophys Acta BBA – Proteins Proteomics 1751, 119–139. 16027053 10.1016/j.bbapap.2005.06.005

[24] Barrow CJ , Yasuda A , Kenny PTM & Zagorski MG (1992) Solution conformations and aggregational properties of synthetic amyloid β‐peptides of Alzheimer's disease: analysis of circular dichroism spectra. J Mol Biol 225, 1075–1093. 1613791 10.1016/0022-2836(92)90106-t

[25] Forneris F , Orru R , Bonivento D , Chiarelli LR & Mattevi A (2009) ThermoFAD, a Thermofluor‐adapted flavin *ad hoc* detection system for protein folding and ligand binding. FEBS J 276, 2833–2840. 19459938 10.1111/j.1742-4658.2009.07006.x

[26] Tan TC , Pitsawong W , Wongnate T , Spadiut O , Haltrich D , Chaiyen P & Divne C (2010) H‐bonding and positive charge at the N(5)/O(4) locus are critical for covalent flavin attachment in *Trametes* pyranose 2‐oxidase. J Mol Biol 402, 578–594. 20708626 10.1016/j.jmb.2010.08.011

[27] Wongnate T , Sucharitakul J & Chaiyen P (2011) Identification of a catalytic base for sugar oxidation in the pyranose 2‐oxidase reaction. ChemBioChem 12, 2577–2586. 22012709 10.1002/cbic.201100564

[28] Kenney WC , Edmondson DE & Seng RL (1976) Identification of the covalently bound flavin of thiamin dehydrogenase. J Biol Chem 251, 5386–5390. 8464

[29] Macheroux P (1999) UV‐visible spectroscopy as a tool to study flavoproteins In Flavoprotein Protocols (Chapman SK & Reid GA, eds), pp. 1–23. Humana Press, Totowa, NJ. 10.1385/1-59259-266-X:1 10494538

[30] Heuts DPHM , Scrutton NS , McIntire WS & Fraaije MW (2009) What's in a covalent bond? On the role and formation of covalently bound flavin cofactors. FEBS J 276, 3405–3427. 19438712 10.1111/j.1742-4658.2009.07053.x

[31] Hassan‐Abdallah A , Bruckner RC , Zhao G & Jorns MS (2005) Biosynthesis of covalently bound flavin: isolation and *in vitro* flavinylation of monomeric sarcosine oxidase apoprotein. Biochemistry 44, 6452–6462. 15850379 10.1021/bi047271x PMC1993914

[32] Schleicher E , Hitomi K , Kay CWM , Getzoff ED , Todo T & Weber S (2007) Electron nuclear double resonance differentiates complementary roles for active site histidines in (6‐4) photolyase. J Biol Chem 282, 4738–4747. 17164245 10.1074/jbc.M604734200

[33] Hecht HJ , Kalisz HM , Hendle J , Schmid RD & Schomburg D (1993) Crystal structure of glucose oxidase from *Aspergillus niger* refined at 2.3 Å resolution. J Mol Biol 229, 153–172. 8421298 10.1006/jmbi.1993.1015

[34] Asensio JL , Ardá A , Cañada FJ & Jiménez‐Barbero J (2013) Carbohydrate–aromatic interactions. Acc Chem Res 46, 946–954. 22704792 10.1021/ar300024d

[35] Bren U & Oostenbrink C (2012) Cytochrome P450 3A4 inhibition by ketoconazole: tackling the problem of ligand cooperativity using molecular dynamics simulations and free‐energy calculations. J Chem Inf Model 52, 1573–1582. 22587011 10.1021/ci300118x

[36] Vasanthanathan P , Olsen L , Jørgensen FS , Vermeulen NPE & Oostenbrink C (2010) Computational prediction of binding affinity for CYP1A2‐ligand complexes using empirical free energy calculations. Drug Metab Dispos 38, 1347–1354. 20413725 10.1124/dmd.110.032946

[37] Shirts MR , Mobley DL & Brown SP (2010) Free energy calculations in structure‐based drug design, pp. 61–86. Structure‐ and Ligand‐Based Approaches, Cambridge University Press, Drug Design.

[38] De Ruiter A & Oostenbrink C (2012) Efficient and accurate free energy calculations on trypsin inhibitors. J Chem Theory Comput 8, 3686–3695. 26593013 10.1021/ct200750p

[39] Ruiz‐Matute AI , Hernández‐Hernández O , Rodríguez‐Sánchez S , Sanz ML & Martínez‐Castro I (2011) Derivatization of carbohydrates for GC and GC–MS analyses. J Chromatogr B 879, 1226–1240. 10.1016/j.jchromb.2010.11.013 21186143

[40] Little JL (1999) Artifacts in trimethylsilyl derivatization reactions and ways to avoid them. J Chromatogr A 844, 1–22. 10399322 10.1016/s0021-9673(99)00267-8

[41] Volc J , Sedmera P , Halada P , Přikrylová V & Daniel G (1998) C‐2 and C‐3 oxidation of d‐Glc, and C‐2 oxidation of d‐Gal by pyranose dehydrogenase from *Agaricus bisporus* . Carbohydr Res 310, 151–156. 10.1016/s0008-6215(00)00167-1 11086703

[42] Wilke T , Schneider M & Kleinermanns K (2013) 1,4‐hydroquinone is a hydrogen reservoir for fuel cells and recyclable *via* photocatalytic water splitting. Open J Phys Chem 03, 97–102.

[43] Fraaije MW , van den Heuvel RHH , van Berkel WJH & Mattevi A (1999) Covalent flavinylation is essential for efficient redox catalysis in vanillyl‐alcohol oxidase. J Biol Chem 274, 35514–35520. 10585424 10.1074/jbc.274.50.35514

[44] Mattevi A (2006) To be or not to be an oxidase: challenging the oxygen reactivity of flavoenzymes. Trends Biochem Sci 31, 276–283. 16600599 10.1016/j.tibs.2006.03.003

[45] Gadda G (2012) Oxygen activation in flavoprotein oxidases: the importance of being positive. Biochemistry 51, 2662–2669. 22432926 10.1021/bi300227d

[46] Baron R , Riley C , Chenprakhon P , Thotsaporn K , Winter RT , Alfieri A , Forneris F , Van Berkel WJH , Chaiyen P , Fraaije MW *et al* (2009) Multiple pathways guide oxygen diffusion into flavoenzyme active sites. Proc Natl Acad Sci USA 106, 10603–10608. 19541622 10.1073/pnas.0903809106 PMC2698890

[47] McDonald CA , Fagan RL , Collard F , Monnier VM & Palfey BA (2011) Oxygen reactivity in flavoenzymes: context matters. J Am Chem Soc 133, 16809–16811. 21958058 10.1021/ja2081873 PMC3203534

[48] Chaiyen P , Fraaije MW & Mattevi A (2012) The enigmatic reaction of flavins with oxygen. Trends Biochem Sci 37, 373–380. 22819837 10.1016/j.tibs.2012.06.005

[49] Braman J (ed.) (2002) In vitro mutagenesis protocols. Humana Press, Totowa, New Jersey.

[50] Kujawa M , Volc J , Halada P , Sedmera P , Divne C , Sygmund C , Leitner C , Peterbauer C & Haltrich D (2007) Properties of pyranose dehydrogenase purified from the litter‐degrading fungus *Agaricus xanthoderma* . FEBS J 274, 879–894. 17227387 10.1111/j.1742-4658.2007.05634.x

[51] Macheroux P , Massey V , Thiele DJ & Volokita M (1991) Expression of spinach glycolate oxidase in *Saccharomyces cerevisiae*: purification and characterization. Biochemistry 30, 4612–4619. 1850628 10.1021/bi00232a036

[52] Pantoliano MW , Petrella EC , Kwasnoski JD , Lobanov VS , Myslik J , Graf E , Carver T , Asel E , Springer BA , Lane P *et al* (2001) High‐density miniaturized thermal shift assays as a general strategy for drug discovery. J Biomol Screen 6, 429–440. 11788061 10.1177/108705710100600609

[53] Wilkinson AJ , Fersht AR , Blow DM & Winter G (1983) Site‐directed mutagenesis as a probe of enzyme structure and catalysis: tyrosyl‐tRNA synthetase cysteine‐35 to glycine‐35 mutation. Biochemistry 22, 3581–3586. 6615786 10.1021/bi00284a007

[54] Fersht A (1999) Structure and mechanism in protein science: a guide to enzyme catalysis and protein folding. Freeman, New York, W.H.

[55] Schmid N , Christ CD , Christen M , Eichenberger AP & van Gunsteren WF (2012) Architecture, implementation and parallelisation of the GROMOS software for biomolecular simulation. Comput Phys Commun 183, 890–903.

[56] Oostenbrink C , Villa A , Mark AE & Van Gunsteren WF (2004) A biomolecular force field based on the free enthalpy of hydration and solvation: the GROMOS force‐field parameter sets 53A5 and 53A6. J Comput Chem 25, 1656–1676. 15264259 10.1002/jcc.20090

[57] Berendsen HJC , Postma JPM , Van Gunsteren WF & Hermans J (1981) Intermolecular Forces, pp. 331–342. Springer, Netherlands.

[58] Amadei A , Chillemi G , Ceruso MA , Grottesi A & Di Nola A (2000) Molecular dynamics simulations with constrained roto‐translational motions: theoretical basis and statistical mechanical consistency. J Chem Phys 112, 9–23.

[59] Berendsen HJC , Postma JPM , van Gunsteren WF , DiNola A & Haak JR (1984) Molecular dynamics with coupling to an external bath. J Chem Phys 81, 3684–3690.

[60] Ryckaert J‐P , Ciccotti G & Berendsen HJ (1977) Numerical integration of the cartesian equations of motion of a system with constraints: molecular dynamics of n‐alkanes. J Comput Phys 23, 327–341.

[61] Tironi IG , Sperb R , Smith PE & van Gunsteren WF (1995) A generalized reaction field method for molecular dynamics simulations. J Chem Phys 102, 5451–5459.

[62] Heinz TN , van Gunsteren WF & Hünenberger PH (2001) Comparison of four methods to compute the dielectric permittivity of liquids from molecular dynamics simulations. J Chem Phys 115, 1125–1136.

[63] Åqvist J , Medina C & Samuelsson JE (1994) A new method for predicting binding affinity in computer‐aided drug design. Protein Eng 7, 385–391. 8177887 10.1093/protein/7.3.385

[64] Åqvist J , Luzhkov VB & Brandsdal BO (2002) Ligand binding affinities from MD simulations. Acc Chem Res 35, 358–365. 12069620 10.1021/ar010014p

[65] Stjernschantz E & Oostenbrink C (2010) Improved ligand‐protein binding affinity predictions using multiple binding modes. Biophys J 98, 2682–2691. 20513413 10.1016/j.bpj.2010.02.034 PMC2877349

[66] Fiehn O , Kopka J , Trethewey RN & Willmitzer L (2000) Identification of uncommon plant metabolites based on calculation of elemental compositions using gas chromatography and quadrupole mass spectrometry. Anal Chem 72, 3573–3580. 10952545 10.1021/ac991142i

[67] Chu DB , Troyer C , Mairinger T , Ortmayr K , Neubauer S , Koellensperger G & Hann S (2015) Isotopologue analysis of sugar phosphates in yeast cell extracts by gas chromatography chemical ionization time‐of‐flight mass spectrometry. Anal Bioanal Chem 407, 2865–2875. 25673246 10.1007/s00216-015-8521-9

[68] Van Den Dool H & Kratz P (1963) A generalization of the retention index system including linear temperature programmed gas—liquid partition chromatography. J Chromatogr A 11, 463–471. 10.1016/s0021-9673(01)80947-x 14062605

[69] Sucharitakul J , Tongsook C , Pakotiprapha D , van Berkel WJH & Chaiyen P (2013) The reaction kinetics of 3‐hydroxybenzoate 6‐hydroxylase from *Rhodococcus jostii* RHA1 provide an understanding of the para‐hydroxylation enzyme catalytic cycle. J Biol Chem 288, 35210–35221. 24129570 10.1074/jbc.M113.515205 PMC3853271

[70] Massey V (1991) A simple method for determination of redox potentials In Flavins and Flavoproteins (Curti B, Zanetti G & Ronchi S, eds), pp. 59–66. Walter de Gruyter, Berlin.

[71] Chaiyen P , Brissette P , Ballou DP & Massey V (1997) Thermodynamics and reduction kinetics properties of 2‐methyl‐3‐hydroxypyridine‐5‐carboxylic acid oxygenase. Biochemistry 36, 2612–2621. 9054568 10.1021/bi962325r

[72] Sucharitakul J , Wongnate T , Montersino S , van Berkel WJH & Chaiyen P (2012) Reduction kinetics of 3‐hydroxybenzoate 6‐hydroxylase from *Rhodococcus jostii* RHA1. Biochemistry 51, 4309–4321. 22559817 10.1021/bi201823c

[73] Clarke WM (1960) Oxidation‐Reduction Potentials of Organic Systems (VJ Shiner Jr.). Wavery Press, Inc., Baltimore pp 248–272.

[74] Einarsdottir GH , Stankovich MT & Tu SC (1988) Studies of electron‐transfer properties of salicylate hydroxylase from *Pseudomonas cepacia* and effects of salicylate and benzoate binding. Biochemistry 27, 3277–3285. 3390431 10.1021/bi00409a023

[75] Massey V , Komai H , Palmer G & Elion GB (1970) On the mechanism of inactivation of xanthine oxidase by allopurinol and other pyrazolo[3,4‐d]pyrimidines. J Biol Chem 245, 2837–2844. 5467924

