# Supplementary material for: Reaction of pyranose dehydrogenase from Agaricus meleagris with its carbohydrate substrates
Source: FEBS J. 2015 Sep 11;282(21):4218–41. doi: 10.1111/febs.13417 (PMC4950071; doi:10.1111/febs.13417)
Supplement: Supplementary file 1 — Table S1. Nucleotide sequences of the primers used. [file FEBS-282-4218-s001.zip › febs13417-sup-0001-supinfo.pdf]

Supplementary material

Reaction of pyranose dehydrogenase from *Agaricus meleagris* with its carbohydrate substrates

Michael M. H. Graf[1], Jeerus Sucharitakul[2], Urban Bren[3,4], Dinh Binh Chu[5,6], Gunda Koellensperger[7], Stephan Hann[5], Paul G. Furtmüller[8], Christian Obinger[8], Clemens K. Peterbauer[1], Chris Oostenbrink[3], Pimchai Chaiyen[9], Dietmar Haltrich[1]

1 Food Biotechnology Laboratory, Department of Food Science and Technology, University of Natural Resources and Life Sciences Vienna (BOKU), Muthgasse 18, 1190 Vienna, Austria.

2 Department of Biochemistry, Faculty of Dentistry, Chulalongkorn University, Henri-Dunant Road, Patumwan, 10330 Bangkok, Thailand

3 Institute of Molecular Modeling and Simulation, University of Natural Resources and Life Sciences Vienna (BOKU), Muthgasse 18, 1190 Vienna, Austria.

4 Laboratory for Physical Chemistry and Chemical Thermodynamics, Faculty of Chemistry and Chemical Technology, University of Maribor, Smetanova 17, 2000 Maribor, Slovenia.

5 Division of Analytical Chemistry, Department of Chemistry, University of Natural Resources and Life Sciences Vienna (BOKU), Muthgasse 18, 1190 Vienna, Austria.

6 School of Chemical Engineering, Department of Analytical Chemistry, Hanoi University of Science and Technology, No 1 Daicoviet, Hanoi, Vietnam.

7 Institute of Analytical Chemistry, Faculty of Chemistry, University of Vienna, Waehringer Straße 42, 1090 Vienna, Austria.

8 Division of Biochemistry, Department of Chemistry, University of Natural Resources and Life Sciences Vienna (BOKU), Muthgasse 18, 1190 Vienna, Austria.

[9] Department of Biochemistry and Center of Excellence in Protein Structure and Function, Faculty of Science, Mahidol University, 10400 Bangkok, Thailand

Corresponding author: Dietmar Haltrich, Department of Food Science and Technology, University of Natural Resources and Life Sciences (BOKU), Muthgasse 18, 1190 Vienna, Austria.

Tel.: +43 1 47654 6140. Fax: +43 1 47654 6251.

E-mail: dietmar.haltrich@boku.ac.at.

Website: http://www.dlwt.boku.ac.at/food-technology/

Table S1 Nucleotide sequences of the primers used

The primers were necessary for removing the *c-myc* epitope of the pPICZ B vector, adding the *Not*I and *Xba*I restriction sites to the 3'- and 5'-ends of the *ampdh* gene, respectively, and for site-directed mutagenesis. Restriction and mutagenesis sites are highlighted in bold, underlined letters.

Name	Sequence (5'–3')
pPICZB-6His-fw	CATCATCATCATCATCATTGAGTTTGTAGCCTTAGACATG
pPICZB-6His-*Xba*I-rv	ATGATGATGATGATG**TCTAGA**AAGCTGGCGGCCGCCGCGGC
*Am*PDH-*Not*I-fw	AAA**GCGGCCGC**ATGCTGCCTCGAGTGACCAAGTTG
*Am*PDH-*Xba*I-rv	TTT**TCTAGA**GTTATAACTCTTTGCTATCAACGC
H103A-fw	GGTGGCTGCAGTACT**GCT**AATGGAATGGTGTACACC
H103A-rv	AGTACTGCAGCCACCCAGGATCTTTGC
Q392A-fw	CCACATATTGAGTTC**GCT**TTTGCACAAATCACCCC
Q392A-rv	GAACTCAATATGTGGTGAATTCTTGCC
Y510A-fw	CTTCAACATTCTCA**GCT**GTGCATGGTGTGGGAACG
Y510A-rv	TGAGAATGTTGAAGATCGCAGG
V511F-fw	CAACATTCTCATAC**TTC**CATGGTGTGGGAACGTTG
V511W-fw	CAACATTCTCATAC**TGG**CATGGTGTGGGAACGTTG
V511-rv	GTATGAGAATGTTGAAGATCGCAGG
H512A-fw	ATTCTCATACGTG**GCT**GGTGTGGGAACGTTGTCG
H512A-rv	CACGTATGAGAATGTTGAAGATCG
H556A-fw	GCTCCGGCCGCA**GCT**ACTCAACTACCTGTTTACGC
H556A-rv	AGT**AGC**TGCGGCCGGAGCATGC
H556N-fw	GCTCCGGCCGCA**AAC**ACTCAACTACCTGTTTACGC
H556N-rv	AGT**GTT**TGCGGCCGGAGCATGC
